# The Effects of a Low Sodium Meal Plan on Blood Pressure in Older Adults: The SOTRUE Randomized Feasibility Trial

**DOI:** 10.3390/nu13030964

**Published:** 2021-03-16

**Authors:** Stephen P. Juraschek, Courtney L. Millar, Abby Foley, Misha Shtivelman, Alegria Cohen, Virginia McNally, Robert Crevatis, Stephen M. Post, Kenneth J. Mukamal, Lewis A. Lipsitz, Jennifer L. Cluett, Roger B. Davis, Shivani Sahni

**Affiliations:** 1 Department of Medicine, Beth Israel Deaconess Medical Center, Boston, MA 02215, USA; courtneymillar@hsl.harvard.edu (C.L.M.); kmukamal@bidmc.harvard.edu (K.J.M.); lipsitz@hsl.harvard.edu (L.A.L.); jlcluett@bidmc.harvard.edu (J.L.C.); rdavis@bidmc.harvard.edu (R.B.D.); shivanisahni@hsl.harvard.edu (S.S.); 2 Harvard Medical School, Boston, MA 02115, USA; 3 Hinda and Arthur Marcus Institute for Aging Research, Hebrew SeniorLife, Roslindale, MA 02131, USA; afole89@gmail.com (A.F.); MishaShtivelman@hsl.harvard.edu (M.S.); alegriacohen@hsl.harvard.edu (A.C.); gmcnally9@gmail.com (V.M.); 4 The Jack Satter House, Revere, MA 02151, USA; RobertCrevatis@hsl.harvard.edu (R.C.); StephenPost@hsl.harvard.edu (S.M.P.)

**Keywords:** sodium, trial, older adults, blood pressure, hypertension

## Abstract

Reduced sodium meal plans are recommended by the Centers of Disease Control to lower blood pressure in older adults; however, this strategy has not been tested in a clinical trial. The Satter House Trial of Reduced Sodium Meals (SOTRUE) was an individual-level, double-blind, randomized controlled pilot study of adults living in a congregate living facility subsidized by the Federal Department of Housing and Urban Development (HUD). Adults over age 60 years ate 3 isocaloric meals with two snacks daily for 14 days. The meal plans differed in sodium density (<0.95 vs. >2 mg/kcal), but were equivalent in potassium and macronutrients. Seated systolic BP (SBP) was the primary outcome, while urine sodium-creatinine ratio was used to measure compliance. Twenty participants were randomized (95% women; 95% white; mean age 78 ± 8 years), beginning in 7 October 2019. Retention was 100% with the last participant ending 4 November 2019. Mean baseline SBP changed from 121 to 116 mmHg with the typical sodium diet (−5 mmHg; 95% CI: −18, 8) and from 123 to 112 mmHg with the low sodium diet (−11 mmHg; 95% CI: −15.2, −7.7). Compared to the typical sodium meal plan, the low sodium meal plan lowered SBP by 4.8 mmHg (95% CI: −14.4, 4.9; *p* = 0.31) and urine sodium-creatinine ratio by 36% (−36.0; 95% CI: −60.3, 3.4; *p* = 0.07), both non-significant. SOTRUE demonstrates the feasibility of sodium reduction in federally mandated meal plans. A longer and larger study is needed to establish the efficacy and safety of low sodium meals in older adults.

## 1. Introduction

Hypertension affects over 85% of older adults [1] and is a contributor to cardiovascular disease [2,3]. Sodium reduction is a central lifestyle strategy to lower blood pressure (BP) for all ages [4]. Nevertheless, 90% of Americans consume an excess of sodium [5,6], and older adults are no exception. Due to mobility concerns that limit food access [7], older adults frequently rely on others for food preparation. One significant source of meals for seniors is the Section 202 supportive housing for the elderly program, operated by the Department of Housing and Urban Development (HUD). This program provides low-cost housing to older adults who are at least 62 years of age and supports over 400,000 housing units for older adults, representing an annual budget of over USD 440 million [8]. Many of these facilities require residents to participate in mandatory meal plans [9] which are often high in sodium to combat age-related declines in taste.

Interventions targeting nutrition services of senior living facilities represent a translational opportunity formally recognized by the Centers of Disease Control and Prevention (CDC) as a critical strategy to improve the health of older adults on a large scale [10]. Reducing the average sodium intake by just 400 mg per day could potentially avert ~28,000 deaths and save USD 7 billion in health care costs annually in the U.S [5]. Nevertheless, some healthcare professionals worry that sodium reduction in older adults might increase orthostatic hypotension and risk of falls [11]. However, there is little evidence to substantiate this concern. In fact, to our knowledge, a reduced sodium meal plan has never been tested in a senior living facility as an intervention in a clinical trial.

The objective of the present study was to determine the feasibility of a 2-week, low sodium meal plan intervention in Section 202 housing. In addition, we examined the effects of the diet on (1) the primary outcome of seated systolic BP (SBP), (2) secondary outcomes, including seated diastolic BP (DBP) and fall risk factors (e.g., a timed-up-and-go test [TUG], standing BP, orthostatic hypotension, orthostatic symptoms), and (3) compliance, based on self-report or measured urine sodium-to-creatinine ratio.

## 2. Methods

The Satter House Trial of Reduced Sodium Meals (SOTRUE) was an investigator-initiated, randomized, double-blind, parallel-arm feeding study conducted in Revere, Massachusetts. The study was sponsored by the Interventional Studies in Aging Center (ISAC) of the Marcus Institute for Aging at Hebrew SeniorLife, a research affiliate of Harvard Medical School. The study protocol was approved by the Institutional Review Board at Hebrew SeniorLife and the trial was registered in clinicaltrials.gov (NCT04074941). The trial was performed in concordance with the Declaration of Helsinki and received ethical approval from the Institutional Review Board (IRB-2019-22) at Hebrew SeniorLife. Each participant provided written informed consent. The first participant was enrolled (randomized) on 7 October 2019; data collection ended 4 November 2019. See Supplementary Table S1 for a schedule of study visits and assessments.

### 2.1. Participants

Eligible participants were residents of Jack Satter House, a congregate living facility, subsidized by the HUD Section 202 housing program. Residents of this facility are independently living and their rent contributes to a mandatory meal plan (dinner, Monday–Friday); however, residents are able to opt-out of their plans for health reasons and have flexibility in choosing food items within the plan. Study participants were required to be age 60 years or older, with a resting SBP of 100–149 mmHg and DBP < 100 mmHg, and on stable BP medications (no recent changes in the past 2 months or anticipated changes during the study period). For further eligibility details see Supplementary Material S1. Participants were recruited via digital advertisements, monthly town meetings, dedicated study informational sessions, study flyers, and telephone calls to participant residences. Interested adults underwent a prescreening medical interview by telephone and two in-person visits prior to randomization.

### 2.2. Intervention

The intervention was provided to all trial participants 16–31 October 2019. Eligible adults were randomized to one of two 2-week meal plans: a low sodium-dense meal plan (sodium density < 0.95 mg/kcal) or a typical sodium-dense meal plan (sodium density > 2 mg/kcal), based on the 75th percentile of consumption among U.S. adults aged 60 years and older [12,13]. The low sodium density was chosen to reflect a reduction in sodium density of about 1.1 mg/kcal (reflecting the high versus low contrast in the DASH-Sodium trial) [14]. Meals were designed to be slightly reduced in carbohydrates to accommodate adults with diabetes with a target range of 45–55% of energy from carbohydrates. Moreover, both meal plans were designed to have equivalent amounts of potassium. The nutrient composition of the two meal plans is shown in Table 1. The meal plans were created by a registered clinical dietitian using the Computrition software platform (West Hills, Lemoore, CA, USA) to include 7 unique days with up to 2 snacks at 3 distinct energy levels: 1750 kcal/day, 2000 kcal/day, and 2250 kcal/day. The kilocalorie level for each participant was based on their estimated energy needs derived from the Mifflin St. Jeor estimator [15], using activity factors from the Godin-Shephard Leisure-Time Questionnaire [16]. Participants completed daily meal tracking surveys to monitor compliance with each meal and to document any non-protocol foods. When it came to main proteins/entrees, attempts were made to use the same products. Since meals were prepared fresh, on-site, recipes were adjusted according to dietitians’ guidance with regards to added ingredients (e.g., sodium). This kept costs similar between diets. To enhance flavor, sodium was replaced with fresh herbs and homemade food stocks.

A permuted block randomization scheme with randomly varying block sizes of 2, 4, or 6 was used in strata of sex. Details of the blocks and assignments were concealed from participants, research staff, investigators, and analytic team. Assignment codes were communicated to the culinary team who had exclusive access to the key linking assignment with participant. Study personnel and investigators were masked until all data collection was completed. The analyst was also masked until the analysis was complete.

### 2.3. Primary Outcome: Seated Systolic Blood Pressure

The primary outcome was seated SBP measured in triplicate (with 30 s between measurements) in the attendance of a research assistant, using an automatic, oscillometric device, (Omron HEM907 XL) after 5 min of seated rest. Participants were asked to empty their bladder and refrain from exercise, food, or caffeine for at least 30 min prior to measurement. The three SBP measurements were averaged within each visit and also across two study visits. For baseline SBP, one BP measurement occurred between 2–3 weeks before feeding and the other occurred the day before feeding. For follow-up SBP, one visit occurred at day 10–12 and the other on day 14–15 of the study.

### 2.4. Secondary Outcomes

Pre-specified secondary outcomes were seated DBP, standing BP, orthostatic hypotension (OH), orthostatic symptoms, a TUG test, body mass index (BMI), urine sodium, urine potassium, urine creatinine, urine sodium-creatinine, and urine potassium-creatinine. For detailed methods see Supplementary Material S2.

### 2.5. Safety, Symptoms, Compliance, and Palatability

We pre-specified outcomes related to safety (falls and allergic reactions), common symptoms related to diet (uncomfortably full, hunger, bloating, constipation, diarrhea, excessive thirst, fatigue, headache, lightheadedness with standing, nausea, and nocturia), compliance, and palatability (see Supplementary Material S2).

### 2.6. Other Covariates

For definitions of other covariates used to characterize our population, see Supplementary Material S2.

### 2.7. Statistical Analysis

We used means and proportions to describe baseline population characteristics. We used an intention-to-treat approach to analyze our primary outcome, seated SBP. Our primary contrast was the low sodium meal plan versus the typical sodium meal plan performed using linear regression models adjusted for baseline seated SBP [17]. To detect the 2-week difference in seated SBP (primary outcome) observed in the DASH-Sodium trial [14] (−9.53 mmHg, standard deviation of 10.46) with a type 1 error of 0.05 and 80% power, we anticipated requiring 40 people (20 in each arm). In addition to aggregate comparisons, we also present within-person changes in SBP from baseline using spaghetti plots.

Changes in secondary outcomes (DBP, TUG test, OH, OH symptoms, BMI, urine sodium and potassium measures) were also compared to baseline and between meal plans (with adjustment for baseline). Urine measures were all log-transformed to address skewed residuals with data presented using geometric means or %-differences. Pre-specified adverse events and change in baseline symptoms scores were compared between meal plans using generalized estimating equation (GEE) regression models with a Huber and White robust variance estimator [18], which assumed an exchangeable working correlation matrix, adjusted for baseline assessments. We used change in baseline symptoms to address non-normally distributed residuals.

Changes in SBP and DBP from baseline were compared with changes in urine sodium and urine potassium from baseline using linear regression adjusted for age, sex, and race. The correlations in changes were also compared using Pearson’s coefficient.

Pre-specified subgroup analyses were performed in strata of sex, baseline hypertension (HTN) medication use, and self-reported diabetic conditions. In exploratory analyses, we also examined strata of calorie adequacy, obesity, and self-reported adherence. Self-reported compliance was based on the responses to the following 2 Likert questions at 1 week and 2 weeks: “How often did you waste or store food because it was too much?” and “How often did you need to supplement foods with non-study foods because it was too little?” A mean score of 1 or 2 was considered compliant. Interaction terms were used to compare strata. In addition, we performed the following sensitivity analyses: on-treatment analysis (excluding the 2 adults who discontinued meals), restricting Omron assessments to the last 2 of 3 BP measurements (reported to be more valid in prior studies) [19], examining SBP as a change from baseline, examining SBP as a repeat measures analysis (versus an average of two visits), and using the SBP measurement from the 14-day follow-up visit alone.

Mean Likert scores for compliance and palatability were estimated according to diet based on the average responses over 1-week and 2-week visits.

All analyses were performed with Stata version 15.1 (Stata Corporation, College Station, TX, USA). Given that this was a pilot study, while a *p*-value of < 0.05 was considered statistically significant, *p*-values ≤ 0.25 were pre-specified in our statistical analysis plan as noteworthy for discussion and further study [20].

## 3. Results

### 3.1. Baseline Characteristics

Participant flow through the study is displayed in Supplementary Figure S1. Of the approximately 285 residents of Jack Satter House, 59 adults expressed interest in participation. We ultimately enrolled 20 participants. The mean age of participants was 78 (SD, 8) years, 95% were women, 95% were white, and 55% reported a history of hypertension. Population characteristics were similar between intervention assignments (Table 2).

### 3.2. Primary Outcome: Seated Systolic Blood Pressure

**Figure 1:**
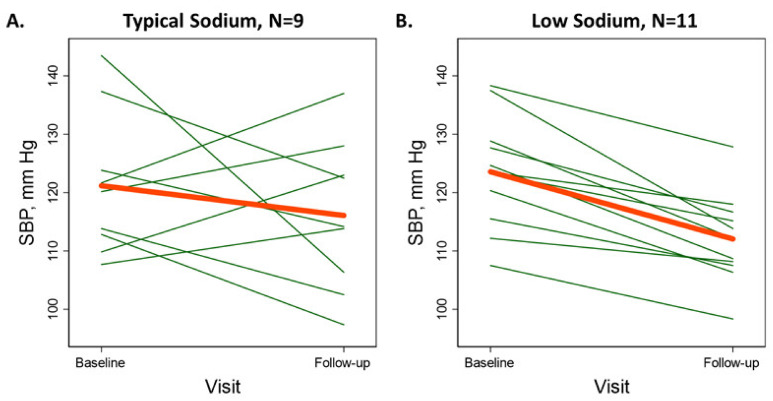
Spaghetti plots of the within-person change in systolic blood pressure (mmHg) between baseline and follow-up (average of days 10 and 14) periods according to (**A**) typical sodium or (**B**) low sodium meal plan assignment.

Among those assigned the typical sodium meal plan, the baseline SBP was 121.1 and 116.1 mmHg after at least 10 days on the meal plan (mean difference −5.0 mmHg; 95% CI: −18.1,8.1; *p* = 0.41; Table 3). Among those assigned the low sodium meal plan, the baseline SBP was 123.5 mmHg and 112.1 mmHg after at least 10 days of the meal plan (mean difference −11.5 mmHg; 95% CI: −15.2,−7.7; *p* < 0.001). The difference in SBP between low versus typical sodium diets was −4.78 (95% CI: −14.41, 4.85; *p* = 0.31). While 5 of 9 participants on the typical sodium plan experienced lower SBP, 11 of 11 had reduced SBP on the lower sodium meal plan (Figure 1).

### 3.3. Secondary Outcomes

Compared to the typical sodium meal plan, the low sodium meal plan non-significantly lowered seated DBP (−2.35 mmHg; 95% CI: −7.97, 3.28; *p* = 0.39). Moreover, low sodium was non-significantly associated with OH (odds ratio 0.28; 95% CI: 0.04, 2.08; *p* = 0.21) and orthostatic symptoms (odds ratio 5.87; 95% CI: 0.28, 123.01; *p* = 0.25). In contrast, low versus typical sodium decreased urine sodium (%-difference −29.7; 95% CI: −47.6, −5.6; *p* = 0.02) and non-significantly decreased urine sodium-creatinine ratio (%-difference −36.0; 95% CI: −60.3, 3.4; *p* = 0.07) (Table 4).

### 3.4. Adverse Events and Symptoms

There were no fall events or allergic episodes. However, 2 participants with baseline diabetes on the typical sodium diet reported elevated blood sugars at home, leading to their decision to discontinue meals (although they did participate in the last study visit). With regard to reported symptoms, the low sodium meal plan was non-significantly associated with diarrhea (*p* = 0.06) and nausea (*p* = 0.07), but was not associated with other symptoms (Supplementary Table S2).

### 3.5. Correlation between BP Changes and Urinary Sodium and Potassium Excretion

Change in SBP and DBP from baseline were positively associated with changes in urine sodium and urine sodium-to-creatinine ratio from baseline (Supplementary Table S3) with Pearson’s *r* coefficients of 0.3 to 0.4. Change in urine potassium was inversely correlated with SBP with a Pearson’s *r* of −0.29.

### 3.6. Pre-stated Subgroups and Sensitivity Analyses

The effects of low versus typical sodium meal plans appeared greater in adults using hypertension medications at baseline (−12.92 mmHg; 95% CI: −25.64,−0.20; *p*-interaction = 0.08), in those for whom their estimated energy needs were exceeded by the mean calories provided (−7.78 mmHg; 95% CI: −18.77,3.21; *p*-interaction = 0.09), and in adults with obesity (−8.78; 95% CI: −22.09,4.52; *p*-interaction = 0.15) (Supplementary Table S4).

In sensitivity analyses, the effect of low sodium on SBP exceeded our significance threshold when restricted to the 18 participants who consumed their meals throughout the trial, when BP was analyzed as a difference in change from baseline (difference in differences), or after restricting Omron assessments to the last two of three BP measurements from each visit (Supplementary Table S5).

### 3.7. Adherence, Palatability, and Blinding

There were 2 participants who discontinued meals on the typical sodium meal plan due to concerns about sugar, resulting in a 59% compliance rate (70% excluding the two who dropped out). Meanwhile, compliance was 71% for the low sodium meal plan. Assessments of palatability, portion appropriateness (meals too large or small), and reasons for non-adherence or consumption of non-protocol foods are in Supplementary Table S6.

After the study was complete, participants were informally asked to guess their assignment: 5 of 9 (64%) assigned the typical sodium and 7 of 11 (56%) assigned the low sodium guessed their assignment correctly. The number that correctly guessed their assignment did not differ from chance (i.e., 50%) or by assignment, suggesting that masking was effective.

## 4. Discussion

In this study, we evaluated the feasibility of a low sodium meal plan intervention to lower BP among older residents of Section 202 housing. Our meal plan significantly reduced urine sodium excretion with strong suggestion for change in SBP during the on-treatment analysis and within those taking hypertension medications at baseline with minimal adverse effects. However, recruitment was challenging, and participants preferred not to continue the meal plans long-term. Given ongoing federal mandates for older adults to participate in Section 202 meal plans, a definitive study on the benefits and risks of sodium reduction in older adults is critically needed to inform dietary strategies that optimize cardiovascular health.

Sodium is a well-established determinant of BP [14]. Furthermore, the TONE trial demonstrated that long-term sodium reduction (i.e., a 24-h dietary sodium intake of 80 mmol [1800 mg] or less on 24-h urine sodium) reduces BP and anti-hypertensive medication utilization among older adults [21]. Based on this evidence, the CDC has recommended low sodium meal plans as a potential strategy to improve BP in older adults [22], a population with widespread hypertension [1] However, dietary interventions differentially affect adults across the range of normal to high BPs, leading some to question whether sodium reduction might increase fall risk. Although our study was not adequately powered to detect a between-diet difference in BP, we note that all eleven participants on the low sodium diet experienced reductions in BP from baseline over a short time period. Furthermore, the meal plan had greater effects among those on hypertension medications, suggesting that a dietary approach may have even greater effects among adults with hypertension versus those without hypertension. A similar observation was made in the DASH-Sodium trial, whereby sodium reduction had greater magnitude effects on adults with higher BP at baseline [23]. A definitive study is needed to substantiate these differential BP-lowering effects in older adults.

There is a paucity of research on the effects of sodium restriction on fall risk factors like orthostatic hypotension. At least a third of our participants reported having a history of a fall that resulted in a fracture, emergency room visit, or hospitalization. The effects of sodium on autonomic function are well-established [24], but may differ in older adults with variable salt sensitivities [25]. We have previously shown that higher sodium intake increased orthostatic lightheadedness in younger adults, but these effects differed by age [26]. While the present pilot study was underpowered, sodium reduction was consistent with reduced risk of OH, but greater risk of having orthostatic symptoms. This disconnect between OH and symptoms has been described in physiology studies and may reflect habitation or cerebral autoregulatory phenomena [27]. Future research should examine fall risk factors, as well as the impact of sodium reduction on fall events among older adults.

One question prior to initiating this study was how to define the typical sodium diet. We used the 75th percentile of sodium density in the U.S. population, but it was unclear how this target (based on nationwide, self-reported intake) would relate to our specific population. Notably, those assigned the typical sodium diet had a non-significant, mean reduction in BP, suggesting that at the very least BP was not worsened in the “typical” group. Thus, the densities and energy levels established in this pilot represent useful guides for subsequent feeding studies.

Our study has limitations. First, we were underpowered to detect a significant between-diet difference in seated BP from sodium reduction at conventional levels of statistical significance. This was expected as we fell short of recruitment goals and enrolled adults without elevated blood pressure. This highlights the challenges of recruitment in this population of older adults, at least with this approach. Future studies should include additional sites to improve the number of enrollees or develop interventions that can be tested at various times. Second, for simplicity we focused on four features of diet (sodium, total energy, energy from carbohydrates, and potassium). It is possible that other features of the diets beyond sodium might contribute to some of the effects observed in our study. Third, our study lasted a short duration. Effects from sodium on BP may take up to 4 weeks [28], while effects on physical function may require even more time to occur. Both the maintenance and magnitude of the sodium effect over time represent an important focus for subsequent research. Fourth, our study was predominantly white, female, and obese, reflecting the underlying characteristics of the community where the study was performed, which limits generalizability. Future studies will likely require more than one location to improve generalizability. Finally, we included adults without elevated BP, who would be expected to experience less of an effect from sodium on BP [23]. However, given that in real-world settings such an intervention would likely impact both normo- and hypertensive adults, we believe including adults without elevated BP is especially important to study the safety of a low sodium dietary intervention. Subsequent studies should include an adequate number of participants to examine both the BP lowering effects as well as the fall risk factors potentially affected by sodium reduction.

Our study has strengths. First, our intervention focused on sodium density based on caloric intake, which is more strongly associated with BP than total sodium per day [29]. Second, we executed a rigorous blinded, randomized, controlled design in a pragmatic, real-world setting, generating data directly applicable to the study’s target population. Third, our study demonstrates the feasibility of a novel setting for a long-term controlled feeding studies on sodium, that is an ethically preferable alternative to some proposed populations (e.g., imprisoned populations) [30].

In conclusion, SOTRUE demonstrates the feasibility of low sodium feeding studies in Section 202 housing facilities in a predominantly white and female population. While our study suggests that sodium reduction improves BP without evidence of increased risk, long-term studies are needed.

## Figures and Tables

**Table 1:** Nutrient Composition of the Two Meal Plans in the SOTRUE Study.

	Typical Sodium		Low Sodium	
	Targeted	Prepared	Targeted	Prepared
**Density target, mg/kcal**	>2.0	*	<0.95	*
**High Calorie**				
Energy, kcal/d	2250	2262	2250	2167
Carbohydrates, %	45–55	52	45–55	50
Sodium, mg/d	>4500	4395	<2100	2096
Potassium, mg/d	~4500	3319	~4500	3233
**Medium Calorie**				
Energy, kcal/d	2000	2091	2000	1977
Carbohydrates, %	45–55	52	45–55	49
Sodium, mg/d	>4000	4071	<1900	1920
Potassium, mg/d	~4000	3223	~4000	3047
**Low Calorie**				
Energy, kcal/d	1750	1861	1750	1842
Carbohydrates, %	45–55	52	45–55	49
Sodium, mg/d	>3500	3589	<1650	1643
Potassium, mg/d	~3500	2953	~3500	2966

* Achieved sodium densities across high, medium, and low Calorie meals for typical sodium were: 1.94, 1.95, and 1.93 mg/kcal, respectively. Achieved sodium densities across high, medium, and low Calorie meals for low sodium were: 0.97, 0.97, and 0.89 mg/kcal, respectively.

**Table 2:** Baseline characteristics by randomized assignment.

	Typical Sodium, N = 9	Low Sodium, N = 11
	Mean (SD) or %	Mean (SD) or %
Age, yr	79.6 (9.4)	76.9 (6.1)
Female, %	100	91
White, %	100	91
Systolic blood pressure, mmHg	121.1 (12.2)	123.5 (9.7)
Diastolic blood pressure, mmHg	62.2 (7.5)	65.3 (11.1)
Hypertension, %	56	55
Hypertension medication use, %	56	64
Self-reported orthostatic hypotension, %	33	9
Measured orthostatic hypotension, %	22	0
Falls fracture/emergency room, %	11	46
Falls & hospitalization	11	9
Months since last fall (among fallers)	28.0 (23.0)	28.7 (34.2)
How often (1 never, 5 every time)		
Concerned about falling	1.7 (0.9)	1.5 (1.2)
Brace yourself after standing to avoid falling	2.7 (1.8)	1.4 (1.2)
Pause before taking a step forward after standing	2.4 (1.9)	1.5 (1.2)
Take time in process of standing to avoid falling	2.1 (1.5)	1.5 (1.2)
Body mass index, kg/m^2^	31.9 (7.9)	32.3 (7.2)
Obesity, %	67	64
Diabetes-related condition *, %	33	27
Medications for pre/diabetes, %	33	18
Cardiovascular disease, %	44	9
Hyperlipidemia, %	56	64
Gastrointestinal conditions (GERD, h. pylori, diverticulitis, colitis, hiatal hernia), %	11	36
History of cancer, %	22	9
Montreal Cognitive Assessment (MOCA) score	23.6 (1.9)	24.3 (3.3)
Physical activity score (scale 0–42) **	22.8 (13.7)	15.9 (15.1)
Timed-up-and-go, sec	13.4 (3.9)	14.0 (6.2)
Current alcohol use, %	44	46
Estimated calorie intake, kcal/d	1785.1 (311.4)	1849.0 (243.6)

Abbreviations: GERD, gastroesophageal reflux disease; * Includes type 2 diabetes, gestational diabetes, prediabetes; ** Godin-Shephard Leisure-Time Physical Activity Score.

**Table 3:** Change in primary and secondary outcomes from baseline.

	Typical Sodium, N = 9				Low Sodium, N = 11			
	Baseline	Follow-up	Difference		Baseline	Follow-up	Difference	
	Mean (SD)	Mean (SD)	Mean (95% CI)	*p*	Mean (SD)	Mean (SD)	Mean (95% CI)	*p*
Seated SBP, mmHg	121.1 (12.2)	116.1 (13.0)	−5.0 (−18.1, 8.1)	0.41	123.5 (9.7)	112.1 (7.6)	−11.5 (−15.2, −7.7)	<0.001 **
Seated DBP, mmHg	62.2 (7.5)	60.4 (8.0)	−1.8 (−7.4, 3.9)	0.50	65.3 (11.1)	60.3 (9.6)	−5.0 (−8.4, −1.6)	0.009 **
Supine SBP, mmHg	130.7 (13.6)	127.8 (20.2)	−2.9 (−19.4, 13.6)	0.70	127.0 (10.1)	120.2 (9.2)	−6.8 (−15.5, 1.9)	0.12 **
Supine DBP, mmHg	69.4 (7.3)	67.3 (12.2)	−2.1 (−10.6, 6.4)	0.59	70.3 (8.6)	67.8 (9.8)	−2.5 (−7.8, 2.9)	0.34
Standing SBP, mmHg	122.9 (14.3)	110.2 (23.5)	−12.7 (−26.7, 1.3)	0.08**	125.2 (14.0)	110.3 (12.8)	−14.9 (−23.0, −6.8)	0.002 **
Standing DBP, mmHg	63.8 (10.3)	61.6 (16.0)	−2.2 (−11.0, 6.5)	0.58	69.6 (11.1)	62.8 (9.0)	−6.8 (−12.0, −1.7)	0.016 **
Timed-Up-and-Go, sec	13.4 (3.9)	13.8 (4.9)	0.3 (−1.3, 1.9)	0.67	14.0 (6.2)	13.8 (5.6)	−0.2 (−1.1, 0.8)	0.70
Orthostatic hypotension, %	22.2	55.6	33.3 (−21.3, 88.0)	0.38	0.0	27.3	27.3 (−8.1, 62.7)	0.25 **
Orthostatic symptoms, %	33.3	11.1	−22.2 (−60.5, 16.1)	0.50	27.3	27.3	0.0 (−34.3, 34.3)	1.00
Body mass index, kg/m^2^	31.9 (7.9)	32.1 (7.9)	0.2 (−0.2, 0.5)	0.29	32.3 (7.2)	32.3 (7.2)	0.0 (−0.2, 0.3)	0.79
	**Baseline**	**Follow-Up**	**%-Difference**		**Baseline**	**Follow-Up**	**%-Difference**	
	Mean (SE) *	Mean (SE) *	**Mean (95% CI)**	***p***	Mean (SE) *	Mean (SE) *	**Mean (95% CI)**	***p***
Urinary sodium	53.0 (0.7)	54.3 (0.5)	2.4 (−21.3, 33.3)	0.84	66.9 (0.5)	46.4 (0.6)	−30.6 (−43.7, −14.4)	0.003 **
Urinary potassium	66.2 (0.3)	64.7 (0.4)	−2.3 (−25.8, 28.5)	0.85	56.8 (0.4)	62.1 (0.4)	9.4 (−17.7, 45.5)	0.50
Urinary creatinine	107.8 (0.5)	96.2 (0.5)	−10.8 (−40.9, 34.8)	0.55	101.4 (0.6)	101.6 (0.6)	0.1 (−14.4, 17.1)	0.99
Urinary sodium-creatinine	0.5 (0.8)	0.6 (0.8)	14.8 (−29.2, 86.1)	0.54	0.7 (0.6)	0.5 (0.7)	−30.7 (−47.9, −7.8)	0.018 **
Urinary potassium-creatinine	0.6 (0.4)	0.7 (0.4)	9.5 (−27.3, 64.9)	0.63	0.6 (0.6)	0.6 (0.5)	9.3 (−10.4, 33.2)	0.35

Abbreviations: SBP, systolic blood pressure; DBP, diastolic blood pressure. * Geometric means. ** While a *p*-value < 0.05 was considered statistically significant, given the small sample of this pilot study, in our statistical analysis plan, a *p* ≤ 0.25 was considered noteworthy for discussion.

**Table 4:** Effects of low versus typical sodium meals on primary and secondary outcomes, N = 20.

	Mean (95% CI)	*p*
Seated systolic blood pressure, mmHg	−4.78 (−14.41, 4.85)	0.31
Seated diastolic blood pressure, mmHg	−2.35 (−7.97, 3.28)	0.39
Supine systolic blood pressure, mmHg	−6.82 (−21.54, 7.91)	0.34
Supine diastolic blood pressure, mmHg	−0.10 (−9.24, 9.04)	0.98
Standing systolic blood pressure, mmHg	−1.68 (−16.33,12.96)	0.81
Standing diastolic blood pressure, mmHg	−3.33 (−12.87, 6.20)	0.47
Timed-Up-and-Go, sec	−0.47 (−2.20, 1.26)	0.58
Orthostatic hypotension, OR	0.28 (0.04, 2.08)	0.21 *
Orthostatic symptoms, OR	5.87 (0.28,123.01)	0.25 *
Body mass index, kg/m^2^	−0.13 (−0.51, 0.25)	0.48
	**%-Difference (95% CI)**	***p***
Urinary sodium	−29.7 (−47.6, −5.6)	0.02 *
Urinary potassium	2.3 (−26.3, 41.9)	0.89
Urinary creatinine	10.8 (−22.2, 58.0)	0.57
Urinary sodium-creatinine	−36.0 (−60.3, 3.4)	0.07 *
Urinary potassium-creatinine	−4.4 (−31.2, 32.9)	0.79
All primary and secondary analyses were adjusted for baseline values		

* While a *p*-value < 0.05 was considered statistically significant, given the small sample of this pilot study, in our statistical analysis plan, a *p* ≤ 0.25 was considered noteworthy for discussion.

## Data Availability

Data are not publically available to protect the privacy of the small number of participants in our study.

## Supplementary Materials

The following are available online at https://www.mdpi.com/2072-6643/13/3/964/s1. Supplementary Methods S1. Eligibility criteria; Supplementary Methods S2. Detailed Methods for Non-primary Outcomes; Supplementary Figure S1. CONSORT diagram; Supplementary Table S1. Table of assessments by study visit; Supplementary Table S2. Effects of low versus typical sodium meals on participant-reported symptoms; Supplementary Table S3. Association of change in blood pressure from baseline with change in urine sodium or urine potassium; Supplementary Table S4. Effects of a low versus typical sodium meal plan on seated systolic blood pressure in pre-specified and exploratory subgroups; Supplementary Table S5. Sensitivity analyses of effects of a low versus typical sodium meal plan on seated systolic blood pressure; Supplementary Table S6. Self-reported dietary adherence and palatability according to dietary assignment; Trial Protocol; Statistical Analysis Plan.

## Abbreviations

BMI	body mass index
CI	confidence interval
DBP	diastolic blood pressure
SBP	systolic blood pressure
OH	orthostatic hypotension
TUG	timed-up-and-go test
