# Supplementary material for: The Effects of a Low Sodium Meal Plan on Blood Pressure in Older Adults: The SOTRUE Randomized Feasibility Trial
Source: Nutrients. 2021 Mar 16;13(3):964. doi: 10.3390/nu13030964 (PMC8002543; doi:10.3390/nu13030964)
Supplement: Supplementary file 1 [file nutrients-13-00964-s001.zip › Supplemental Material/Final protocol.pdf]

SOTRUE Study

Satter hOuse Trial of Reduced sodium mEals

Protocol

Version 1.8

29th October 2019

Marcus Institute for Aging Research
Hebrew SeniorLife
HARVARD MEDICAL SCHOOL AFFILIATE

The Effect of Sodium Reduction on Blood Pressure and Physical Function in Older Adults [SOTRUE]

Study Chairman or Principal Investigator:

Shivani Sahni, PhD

Director, Nutrition Program and Associate Scientist, the Hinda and Arthur Marcus Institute for Aging Research, Hebrew SeniorLife, Assistant Professor of Medicine, Beth Israel Deaconess Medical Center, Harvard Medical School

Supported by:

Interventional Studies in Aging Center, Marcus Institute for Aging Research, Hebrew SeniorLife

Pilot Grant

Study Intervention Provided by: N/A

Sponsor of IND (IDE): N/A

Version 1.8
10/29/2019

SUMMARY OF PROTOCOL VERSIONS AND CHANGES

Version 1.0

- Original version approved by the Marcus Institute, Hebrew SeniorLife Review Board on May 13th, 2018.

Version 1.1

- Changes to consent, recruitment, protocol on June 28, 2019.

Version 1.2

- Changes to protocol approved on July 26th, 2019.

Version 1.3

- Minor changes to previously approved forms approved on August 23rd, 2019.

Version 1.4

- Changes to the recruitment documents approved on August 28th, 2019.

Version 1.5

- Changes in the protocol on September 30th, 2019.

Version 1.6

- Changes in the protocol on October 23rd, 2019.

Version 1.7

- Approval of the incident report on October 24th, 2019.

Version 1.8

- Submission of protocol and final statistical analysis plan to the IRB on October 29th, 2019.

TABLE OF CONTENTS

Page

TABLE OF CONTENTS **1**

SYNOPSIS **4**

Objectives 5
Design and Outcomes 5
Interventions and Duration 6
Sample Size and Population 6

1 Study Objectives **10**

1.1 Primary Objective 10
1.2 Secondary Objectives 10

2 BACKGROUND AND RATIONALE **100**

2.1 Background on Condition, Disease, or Other Primary Study Focus 100
2.2 Study Rationale 11

3 STUDY DESIGN **11**

4 SELECTION AND ENROLLMENT OF PARTICIPANTS **12**

4.1 Inclusion Criteria 12
4.2 Exclusion Criteria 12
4.3 Study Enrollment Procedures 13

5 STUDY INTERVENTIONS **14**

5.1 Interventions, Administration, and Duration 14
5.2 Handling of Study Interventions 14
5.3 Concomitant Interventions N/A 15
5.4 Adherence Assessment 15

6 STUDY PROCEDURES **15**

6.1 Schedule of Evaluations 16
6.2 Description of Evaluations 177
6.2.1 Pre-screening and Screening Evaluation 177

6.2.2 Enrollment, Baseline, and/or Randomization 178
6.2.3 Follow-up Visits 18
6.2.4 Completion/Final Evaluation 19

6.3 Description of Evaluations 19

7 SAFETY ASSESSMENTS 19

7.1 Specification of Safety Parameters 19

7.2 Methods and Timing of Assessing, Recording, and Analyzing Safety Parameters...... 19

7.3 Adverse Events and Serious Adverse Events 20

7.3 Reporting Procedures 21

7.4 Safety Monitoring 22

7.5 Adverse Events and Serious Adverse Events 22

7.6 Reporting Procedures 23

8 INTERVENTION DISCONTINUATION 23

9 STATISTICAL CONSIDERATIONS 24

9.1 General Design Issues 23

9.2 Sample Size and Randomization 23
9.2.1 Treatment Assignment Procedures 23

9.3 Interim analyses and Stopping Rules 24

9.4 Outcomes 24
9.4.1 Primary outcome 244
9.4.2 Secondary outcomes 244

9.5 Data Analyses 245

Sensitivity analyses 255

Analyses of other outcomes 256

Safety outcomes 267

10 DATA COLLECTION AND QUALITY ASSURANCE 267

10.1 Data Collection Forms 267

10.2 Data Management 277

10.3 Quality Assurance 278
10.3.1 Training 278
10.3.2 Quality Control Committee 288
10.3.3 Protocol Deviations 288
10.3.4 Monitoring 29

11 PARTICIPANT RIGHTS AND CONFIDENTIALITY 28

11.1 Institutional Review Board (IRB) Review 28

11.2 Informed Consent Forms 28

11.3 Participant Confidentiality 29

11.4 Study Discontinuation... .29

12 ETHICAL CONSIDERATIONS.. 29

13 COMMITTEES.. 300

14 PUBLICATIONS AND RESEARCH FINDINGS.. 300

15 REFERENCES... 311

SYNOPSIS

Elevated blood pressure (BP) affects virtually all adults over the age of 65 years in the United States and is a risk factor for both cardiovascular events and falls, which are common causes of long-term disability and premature death. Many older adults have limited mobility and experience food insecurity, relying on others for food preparation. Further, meals prepared for older adults are often high in sodium to overcome age-related declines in taste. Because of this, the Centers for Disease Control declared reduced-sodium meal plans in congregate living facilities a priority to combat the hypertension epidemic among older adults. However, many dietitians and health practitioners worry that low sodium in older adults could result in orthostatic hypotension (OH), causing impairments in physical function and consequently falls. Beyond BP there is evidence that sodium intake directly influences balance, seated-to-standing transitions, and gait speed. However, to date there are no clinical trials of the effects of sodium reduction on physical function.

We intend to perform an individual level randomized clinical trial at a community-based, congregate senior living facility that compares a reduced sodium meal plan (<0.9 mg per kcal of energy intake) to their typical meal plan (~2 mg per kcal of energy intake). This proposal has the following aims: (1) to determine the short-term effects of sodium reduction on BP regulation and OH, a hypothesized mechanism by which sodium intake might affect physical function; (2) to determine the short-term effects of a reduced sodium diet on physical function; and (3) to establish the premise for a longer-term trial by examining diet compliance, tolerability, and palatability. Ultimately, our two-tailed hypothesis ensures a high impact result long-term, since no effect on physical function would reinforce the safety of sodium reduction for cardiovascular disease prevention, while a positive relationship would reinforce a cautious approach to sodium reduction in this population. Beyond our scientific aims, our proposal will result in the development of a reduced sodium meal plan, using the leading food industry design platform, which can be readily implemented in a standardized fashion at long-term, senior care facilities throughout the country.

This pilot proposal will inform a clinical trial of sodium reduction in the prepared meals of semi-independent older adults. By demonstrating the impact of sodium reduction on fall risk factors like physical function and OH, our study will provide pertinent and timely guidance as to how sodium content in meals prepared for seniors impacts risk for clinical events that have long-term implications for independence.

Study Title: The Effect of Sodium Reduction on Blood Pressure and Physical Function in Older Adults (SOTRUE)

Objectives

Specify the primary and secondary objectives.

Virtually all older adults have hypertension, a known contributor to both cardiovascular disease and falls. The Centers of Disease Control has advocated for low sodium, senior meal plans as a strategy to lower BP.(1) However, the long-term effects of low sodium intake on physical function are largely unknown in older adults. Our pilot proposal for a randomized feeding study of a low sodium meal plan in a community-based, congregate senior living facility, will not only establish the premise for a definitive long-term trial on sodium reduction and physical function in older adults, but further contribute to a translatable, standardized menu that can be implemented *en masse* at senior living facilities throughout the U.S. Our *long-term goal* is to discover dietary interventions that enhance physical function as adults age. The *objective of this proposed pilot study* is to determine the feasibility of an individual-level, randomized feeding study that examines the impact of sodium reduction on BP regulation among semi-independent, older adults. Our *central hypothesis* is that sodium reduction will reduce seated BP without causing OH. Improved BP regulation will enhance circulatory function, efficiency of dynamic transitions from seated to standing positions, and ultimately a lower risk of falls.

Primary Aim 1: To determine the effects of a low sodium (<0.9 mg per kcal of energy intake), 2-week meal plan compared with a usual meal plan (average sodium ~2 mg per kcal of energy intake) on seated BP among independently living older adults.

Hypothesis Aim 1: *Compared with the usual meal plan, a reduced sodium meal plan will lower seated BP in older adults after 2 weeks.*

Feasibility Aim 1: To evaluate the recruitment experience, meal cost, meal delivery logistics, and compliance with and tolerability of the meal plan (urine sodium and palatability questionnaires).

Feasibility Aim 2: To determine effect size (variance) of secondary outcomes: standing BP, OH (standing minus seated BP), orthostatic symptoms, and a timed up and go test (TUG).

Design and Outcomes

This study is an individual-level, parallel, randomized clinical trial. Eligible participants will be randomized to low versus usual sodium meal plans for two weeks. Assessments will be made at the in-person baseline visit, one week telephonic interview and 2 week in person follow-up visit. This proposal will be executed in collaboration with Dietetic Services of the Jack Satter House (Revere), which is equipped with an industry-grade kitchen, chefs, and in-person dining as well as delivery services that collectively serve 255 residents. Further, the facility has private space for interviews and assessments. We plan to recruit 40 individuals (75% women).

Interventions and Duration

Interventions for this study include:

1. A low sodium diet
2. A usual sodium diet

A participant will be on one of these two intervention arms for a period of 2 weeks. The last study visit for each participant will occur on the last day of their study diet intervention.

Sample Size and Population

Study participants will include residents of Jack Satter House in Revere who are aged 60 years and older. Forty men and women will be enrolled in this study. Participants will be randomized using a computer-generated randomization scheme with varying sized blocks to 1 of 2 sequences in a 1:1 ratio such that approximately half of participants will undergo the low sodium diet intervention first or usual sodium diet intervention.

STUDY TEAM ROSTER

Principal Investigator: Shivani Sahni, PhD

Marcus Institute for Aging Research, Hebrew SeniorLife
B1316
1200 Centre Street
Boston, MA 02131
617-971-5382 (P)
617-971-5339 (F)
ShivaniSahni@hsl.harvard.edu

Main responsibilities/Key roles: Principle investigator, study oversight and project management (maintaining budget, IRB approvals and compliance, clinical trial registration and amendments), development of the statistical analysis plan, REDCap database, training and supervision of study staff, and data collection. Assisting Dr. Juraschek in data analysis, interpretation and manuscript writing.

Co-Investigators: Lewis Lipsitz, MD

Marcus Institute for Aging Research, Hebrew SeniorLife
B1312
1200 Centre Street
Boston, MA 02131
617-971-5318 (P)
617-971-5339 (F)
lipsitz@hsl.harvard.edu

Main responsibilities/Key roles: Co-Investigator and study physician

Co-Investigators: Stephen P. Juraschek, PhD, MD

Assistant Professor, Harvard Medical School
Beth Israel Deaconess Medical Center
Division of General Medicine, Section for Research
330 Brookline Avenue, CO-1309, #216
Boston, MA 02215
Phone: 617.754.1416
Fax: 617.754.1440
sjurasch@bidmc.harvard.edu

Main responsibilities/Key roles: Study physician, development of the protocol and REDCap database, training and supervision of study staff, screening, review of eligibility and safety assessment, data collection, data analysis, and interpretation and manuscript writing.

Co-Investigators: Jennifer L. Beach, MD

Healthcare Associates
Beth Israel Deaconess Medical Center-East
330 Brookline Avenue, Shapiro 6, North Suite
Boston, MA 02215
617-754-9600 (P)
617-667-8665 (F)
jlbeach@bidmc.harvard.edu

Main responsibilities/Key roles: Hypertension expert, study physician, screening, review of eligibility and safety assessment

Co-Investigators: Kenneth Mukamal, MD, MPH

Beth Israel Deaconess Medical Center
General Medicine, CO-1309
330 Brookline Avenue
Boston, MA 02215
617-667-4476 (P)
617-754-1440 (F)
kmukamal@bidmc.harvard.edu

Main responsibilities/Key roles: Co-Investigator and study physician

Co-Investigators: Roger Davis, DSc

Associate Professor of Medicine (Biostatistics)
Beth Israel Deaconess Medical Center
Department of Medicine

Beth Israel Deaconess Med Center
General Medicine, CO-1309
330 Brookline Ave
Boston MA 02215
617-754-1406 (P)
617-754-1440 (F)
rdavis@bidmc.harvard.edu

Main responsibilities/Key roles: Biostatistician

Co-Investigators: Alegria Cohen, MS, RD, LDN

Lead Clinical Dietitian
Hebrew Rehabilitation Center
1200 Centre Street
Roslindale, MA 02131
617-363-8245
AlegriaCohen@hsl.harvard.edu

Main responsibilities/Key roles: Recruitment, menu development and nutrient analysis in Computrition software

Co-Investigators: Virginia McNally, MS, RDN, LDN

Clinical Dietitian
Hebrew Rehabilitation Center
1200 Centre St
Roslindale MA 02131
617-363-8000
Gmcnally9@gmail.com

Main responsibilities/Key roles: Recruitment, menu development and data collection

Co-Investigators: Abby Foley

Research Associate II
Hebrew SeniorLife
1200 Centre St
Roslindale MA 02131
617-971-5475 (P)
abbyfoley@hsl.harvard.edu

Main responsibilities/Key roles: Recruitment, data collection, data entry, order supplies, communicate with laboratory regarding sample acquisition, respond to participants and escalate any medical, safety, or eligibility concerns to a study physician.

Co-Investigators: **Courtney Millar, PhD**

Postdoctoral Fellow
Hebrew SeniorLife
1200 Centre St
Roslindale MA 02131
617-971-5531 (P)
617-971-5339 (F)
courtneymillar@hsl.harvard.edu

Main responsibilities/Key roles: Pre-screening, administer the Timed Up and Go test, and several study questionnaires to participants, participate in recruitment, data collection, data entry, and some data analysis, cross-train with the study coordinator to provide coverage, assume project management responsibilities as delegated by the PI, including maintenance of study dashboard for reports.

1 STUDY OBJECTIVES

1.1 Primary Objective

Primary Aim 1: To determine the effects of a low sodium (<0.9 mg per kcal of energy intake), 2-week meal plan compared with a usual meal plan (average sodium ~2 mg per kcal of energy intake) on seated BP among independently living older adults.

Hypothesis Aim 1: *Compared with the usual meal plan, a reduced sodium meal plan will lower seated BP in older adults after 2 weeks.*

1.2 Secondary Objectives

Feasibility Aim 1: To evaluate the recruitment experience, meal cost, meal delivery logistics, and compliance with and tolerability of the meal plan (urine sodium and palatability questionnaires).

Feasibility Aim 2: To determine effect size (variance) of secondary outcomes: standing BP, orthostatic hypotension (OH, standing minus seated BP), orthostatic symptoms, and a timed up and go test (TUG).

2 BACKGROUND AND RATIONALE

2.1 Background on Condition, Disease, or Other Primary Study Focus

Significance
Hypertension is rampant among older adults and is a major contributor to cardiovascular disease, falls, and early death. Healthy diet is a critical determinant of BP, and decline in diet quality is a common precursor to decline in global health. High sodium intake contributes to high blood pressure (BP), nevertheless, *9 of 10 Americans consume an excessive amount of dietary sodium each day*.(2) Older adults with mobility limitations rely on others to prepare their foods, which are often high in sodium to combat age-related declines in taste. Efforts to reduce sodium in senior meal plans have been stymied by conflicting evidence about its health effects, summarized below.

1. **Both hypertension and OH are fall risk factors.** Uncontrolled high BP is a potent risk factor for falls among older adults.(3) Age-related hypertension results from vascular stiffness, decreased baroreceptor sensitivity, and altered sodium metabolism, contributing to low BP with changes in posture, called OH.(4, 5) These acute drops in BP impair physical function. In our own studies, OH was associated with postural light-headedness, dizziness, and falls.(6-8) In other studies, OH was strongly associated with impaired static (e.g. balance upon standing) (9) and dynamic physical performance (e.g. chair stands, TUG, and 4m gait speed).(10, 11)

2. **Sodium is recommended to treat OH, but also worsens hypertension.** While multiple studies have demonstrated improved orthostatic tolerance with sodium supplementation,(12-15) a 2017 consensus statement highlighted a need for long-term evidence, stating that the "long-term risks associated with greater salt intake…need to be weighed against the short-term risks

of…OH resulting in fall injury…the long-term risks of high sodium diets in individuals with OH have not been well studied."(16, 17)

3. Sodium reduction lowers BP with unknown effects on physical function. Intensive pharmacologic treatment to lower BP reduces risk of OH without increasing risk of falls long-term.(18-20) Moreover, the Trial of Nonpharmacologic Interventions in the Elderly (TONE) study, established sodium reduction as an efficacious, non-pharmacologic intervention to lower BP in older adults.(21) Whether reduced sodium reduced OH, improved physical function, or prevented falls was not reported. Nevertheless, several studies observed that long-term sodium reduction increased orthostatic tolerance in rats (22) and had no effect on orthostatic tolerance in astronauts.(23) In data from the DASH-Sodium trial,(24) we found that reduced sodium in the context of a healthy diet, actually decreased orthostatic light-headedness.(25)

2.2 Study Rationale

Interventional studies supporting sodium reduction have largely been performed in middle-aged adults. Furthermore, sodium reduction is controversial in older adults, (26) due to concerns about OH, which is highly prevalent among older adults and potentially worsened by low sodium. This debate is perpetuated by observational studies that have described a higher risk of mortality among adults consuming a low sodium diet. In contrast, multiple studies have shown that higher sodium consumption increases BP and causes lightheadedness. Furthermore, trials of BP management have demonstrated that lower BP decreases risk of OH without increasing the risk of falls. Given the controversies surrounding sodium reduction, there has been a call for a definitive clinical trial from institutions worldwide, including: the Institutes of Medicine, the World Heart Federation, the European Society of Hypertension, and the European Public Health Association.(26) One of the challenges facing prior studies of sodium reduction is achieving a controlled environment, (21, 27, 28) especially among free living people. This has led some experts to suggest a population of prisoners, nursing home residents, or military personnel as ideal for a sodium trial.(26) However, ethical concerns, confounding medical conditions, or physical exertion requiring higher sodium are concerns in these populations. ***Our approach of recruiting older adults, who participate in a meal plan of a congregate housing facility of HSL overcomes the above limitations, creating a controlled environment with the opportunity for a long-term intervention.***

3 <u>STUDY DESIGN</u>

- This is a placebo-controlled, double-blinded randomized controlled trial.
- Primary outcome includes seated blood pressure and secondary outcomes include orthostatic hypotension and timed up-and-go test.
- The study includes two arms (1) Low sodium meal plan and (2) Usual sodium meal plan. Forty participants will be enrolled in the study.
- Study population includes older men and women residing in the Jack Satter House, a congregate housing facility in Revere. Approximate duration of enrollment period and follow-up (specify individual participant vs. entire trial)

- After baseline data collection, participants will be randomized in a 1:1 ratio to 1 of 2 meal plans: a usual meal plan (this is the current meal plan with an average sodium intake of 2g per kcal of energy intake) or a low sodium meal plan (<0.9 g per kcal of energy intake similar to levels recommended by the American Heart Association recommendations). Both normotensive and hypertensive individuals will be recruited. Hypertension treatment will be considered as a pre-specified stratification variable.
- Urine will be collected from a random void at the baseline and follow up visits to measure sodium, potassium, and random creatinine. The samples will be sent to Quest Diagnostics Laboratories for analysis to measure excretion of these minerals pre and post exposure to the study diet. For urinary sodium the methodology used is ion selective electrode, with a reference range of 28 to 272 mmol/L. For urinary potassium sodium the methodology used is ion selective electrode, with a reference range of 12-129 mmol/L. For random urinary creatinine the methodology is kinetic colorimetry with a reference range for males of 20-320 mg/dl and females of 20-275 mg/dl.
- Quest Diagnostics is a nationally recognized CLIA certified laboratory capable of accurate and reproducible results on the analyses above. Reports are available electronically and will be uploaded directly into the statistical software at HSL via a CSV file.

4 SELECTION AND ENROLLMENT OF PARTICIPANTS

4.1 Inclusion Criteria

Participants must meet all of the inclusion criteria to participate in this study:

- Residents of Jack Satter House, a congregate living facility in Revere, MA. Residents must be present during the study intervention period to receive the study food.
- Age ≥60 years
- Resting systolic blood pressure 100-149 mm Hg and diastolic blood pressure <100 mm Hg
- Stable BP medications (no recent or intended changes)

4.2 Exclusion Criteria

All candidates meeting any of the exclusion criteria at baseline will be excluded from study participation.

- Age<60 or >100 years
- Physical inability to do a timed up and go test
- Standing systolic blood pressure <90 mm Hg

- Seated BP outside these ranges: SBP: 100-149 mm Hg AND DBP: ≥100 mm Hg
- High blood pressure on unsteady medication dose for the last two months
- Terminal or mental illness
- Active heart disease defined as hospitalization in the past 3 months
- Active inflammatory bowel disease, malabsorption, or history of major gastrointestinal surgery
- Active cancer or cancer treatment (exception maintenance therapy for remission)
- Active kidney dialysis or history of kidney transplant
- Plan to leave Satter house in next three months
- Significant food allergies or dietary supplements that would interfere with diet adherence
- Unwillingness to comply with the diet
- Unable to give informed consent
- MOCA test score <18

4.3 Study Enrollment Procedures

<u>Participant recruitment:</u> We plan to recruit participants in several ways:

- We will distribute flyers and other recruitment materials in the dining and recreational areas of the Satter House with staff discussing the program with residents.
- Initial engagement meetings have demonstrated substantial interest among residents. Therefore, we plan to have engagement meetings every week.
- We plan to set up an information table with study flyers outside the resident cafeteria to provide study information and increase recruitment.
- We will advertise the study details (information in the flyer) via voice friend (tele-messaging) system at Satter House as well as television. We anticipate ~75% of residents will have an eligible BP.
- Direct phone calls to the residents actively participating in the Jack Satter House meal plan.

<u>Procedures for documentation of screened participants who were not enrolled:</u> During pre-screening phone calls and in-person screening visits, participants will be provided with the study information and data will be collected related to their medical records and medication list. Any participant deemed ineligible will be reviewed by the study physician Dr. Stephen Juraschek or Dr. Jennifer Beach. Those who were screened but not eligible or unwilling to

participate will be documented in the screening log maintained by the research assistant Ms. Abby Foley.

Consent Procedures: Using the current IRB approved consent form the research assistant or physician will meet with the participant in a quiet, private location in Jack Satter House. The entire consent document will be reviewed with the participant, ensuring the participant has ample time to ask questions and understands the information. If requested by the participant (due to visual difficulty or lower literacy) the consent will be read to the participant. Persons lacking the capacity to provide informed consent for themselves are ineligible for SOTRUE.

The SOTRUE consent is all or none, if the participant refuses any parts or procedures of the study they may not enroll. If the participant decides to enroll they will sign and date the consent form. The study team member will also sign and date the form, and annotate the consent form with the participant's study ID number. A copy of the signed document will be offered to the participant, and the original consent form will be stored in the locked research file, accessible to the research team only.

Randomization procedure: After baseline data collection, participants will be randomized in a 1:1 ratio to 1 of 2 meal plans: a usual meal plan or a low sodium meal plan. Randomization will be achieved using a web-based, password-protected, randomization software. While trial participants will know their intervention assignments, investigators and all study staff involved in baseline and follow-up data collection will be masked to participants' randomization assignments. Study staff involved in administering the dietary intervention and assessing compliance will be masked to participants' BP measurements. We will recruit the entire population cohort prior to initiating the intervention.

5 STUDY INTERVENTIONS

5.1 Interventions, Administration, and Duration

Study interventions consist of two diet options (one usual sodium and one reduced sodium) for a two week period, during which participants will be asked to consume only the study diet provided. The study diets will be administered as described below. All participants will provide daily feedback on their consumption via a daily food log. Any issues of food intolerance or other possible adverse events (e.g. falls) will be reported to the study physicians for follow-up.

5.2 Handling of Study Interventions

Diet and menu development for both lower sodium and usual sodium diets was done by the study dietitians, keeping the diets similar in types of foods and macronutrient content. Diet selections are also isocaloric, based on the Mifflin St. Jeor calculation for each individual's energy expenditure and BMI.

Ingredients for the dietary intervention will be procured by Mr. Robert Crevatis, head chef at Jack Satter House and Mr. Misha Shtivelman, director of the culinary and nutrition services at Hebrew SeniorLife and Jack Satter House. The ingredients will be stored in the kitchen at the

Jack Satter House where chef Crevatis will prepare all the meals each day. Cost of these meals will be paid by the funds from the study and have been subsidized by the department of culinary and nutrition services. During the intervention period, each day chef Crevatis will prepare breakfast, a boxed lunch a two snacks that will be delivered to the participants at their apartment by chef Crevatis and dining room manager Ms. Karen Hagen. Dinner will be prepared separately and will either be delivered to the study participant's apartment or will be picked up in a box with an option of consuming the dinner in the dining room.

Participants will complete a daily food log, during the 2 week intervention period, and palatability questionnaires at study visits, to track compliance with and tolerability of the diet. Participants are blinded to their diet randomization and are asked not to discuss their study food with others during the course of the intervention.

Meals for both the study arms will be kept as similar as possible except for the sodium content.

Based on participant's feedback, the protocol was changed and any participant who reported not consuming their meals via daily food logs were contacted to confirm if their meal portions were adequate. This information was also collected at the 1-week telephone visit for all participants. Portion sizes were altered based on participant's preference. Furthermore, as a safe guard, participants who reported to be diabetic were contacted and their portion sizes and or carbohydrate levels of their meals were adjusted based on participant preference.

5.3 Concomitant Interventions N/A

5.4 Adherence Assessment

Participants will complete a daily food log, during the 2 week intervention period, and palatability questionnaires at study visits, to track compliance with and tolerability of the diet. Participants will be asked to make every effort to consume the study diet in its entirety, or if unable to do so to eat some of each item in each meal. Participants will be asked not to consume any non-study foods during the study intervention, but if they do, to include these foods in their daily diet log.

6 STUDY PROCEDURES

6.1 Schedule of Evaluations

Assessment	Prescreening (by phone)	Screening: Visit-1 (Day-14 to Day -1)	Baseline, Enrollment, Randomization: Visit 2 (Day 0)	Run In Visit 3 (Day -1 to Day -2)	Treatment Visit 4 (Day 7)	Treatment Visit 5 (Day 10-13)	Follow-up: Final Visit 6 (Day 14)
Medical History	X						
MoCA		X					
Seated blood pressure alone		X		X		X	X
Orthostatic hypotension		X					X
Informed Consent		X					
Demographics		X					
Physical Activity		X					
Medications		X	X				X
Height		X					
Weight		X	X				X
TUG			X				X
Urine collection			X				X
Compliance and Palatability					X		X
Symptoms/Adverse Events			X		X		X
Compliance Calendar			each day of feeding -------------------- >				

6.2 Description of Evaluations

6.2.1 Pre-screening and Screening Evaluation

These evaluations occur to determine if the candidate is eligible for the study.

Consenting Procedure
In the current study there are two consent processes, the first occurs at the prescreening visit, when the study research assistant (RA), postdoctoral fellow, or study physician reads the prescreening script to the person and asks them if they are willing to be screened for eligibility to enroll in the study. If they verbally consent then additional data is obtained at both prescreening and screening visits and kept in a locked file until it is determined if the person qualifies for enrollment. The person is given the RAs contact information and encouraged to call with any questions.

At the second half of the screening visit (BV1a) a written consent is obtained by the study research assistant. A copy of the signed consent is provided to the participant. Documentation of signed consent is added to the consent log, which is checked at each visit. If the person declines to consent or is deemed ineligible, the initial information collected will be destroyed.

Residents of Jack Satter House may attend weekly educational sessions lead by members of the study team onsite. These sessions provide information about the study, as well as additional health education around the topics related to the study. Once enrolled in the study participants have access to the RA, study dietician, Study MD, and PI for any questions or concerns.

In the event that the study had a new consent form all currently active participants would be reconsented with the new consent form.

Study research staff or physicians will obtain consent. If the participant requests (due to visual difficulty or lower literacy) the consent will be read to the participant. Participants will be educated in the aims of the study and its implementation. They will have access to speak with members of the study team for any questions concerning the study.

Screening

Visit 1. [Screening (SV) and initial baseline visit (BV1a)]: Eligible participants will be undergo written informed consent. Questionnaire based information on demography, self-reported physical activity and medication list will be obtained for eligible participants as part of the initial baseline visit. Assessments of orthostatic hypotension, MoCA and seated blood pressure will be done at this visit to check for eligibility of the participant. Weight and height will be measured to calculate targeted caloric intake. This information will be needed in advance to prepare meals for the participants.

6.2.2 Enrollment, Baseline, and/or Randomization

Enrollment

For this study enrollment date was defined as the date all of the screening criteria were met and the individual agreed to participate.

Baseline Assessments
For participants who have successfully been screened for eligibility and are enrolled into the study, baseline assessments will be performed against which to measure the study outcome. Run-In visit and baseline visit are described below.

- ***Visit 2 [Randomization/baseline visit (RZ/BV1b)]:***
  - *Weight*
  - *Timed up and go test*
  - *Urine specimens*
  - *Symptoms and adverse events associated with sodium and orthostatic hypotension (headache, lightheadedness, dizziness, fatigue, etc.)*

- ***Visit 3 [Run-In Visit, 1-2 days before meal delivery starts]:***
  - *Seated blood pressure*
  - *Weight*

Randomization
Randomization will precede intervention administration. After baseline data collection, participants will be randomized in a 1:1 ratio to 1 of 2 meal plans: a usual sodium meal plan or a low sodium meal plan. All participants will be randomized at least 1-2 days before the initiation of the study intervention.

6.2.3 Follow-up Visits

- ***Visit 4 [Telephone Visit (TV), Day 7]:***
  - *Diet adequacy for caloric needs*
  - *Compliance*
  - *Palatability*
  - *Symptoms and Adverse Events*

- ***Visit 5 (Intermediate Visit), Day 11-13:***
  - *Seated blood pressure*

6.2.4 Completion/Final Evaluation

- ***Visit 6 (Follow-up Visit), Day 14:***
  - *Seated blood pressure*
  - *Weight*
  - *Timed up and go test*
  - *Urine specimens*

- *Symptoms and adverse events associated with sodium and orthostatic hypotension (headache, lightheadedness, dizziness, fatigue, etc.)*

6.3 Description of Evaluations

<u>*Urinary sodium and potassium and creatinine excretion.*</u> Spot urine collections, urinary sodium (Na), potassium (K), and creatinine excretion will be used to estimate group compliance with dietary aspects of the intervention (i.e., sodium excretion for salt intake and potassium excretion for fruit and vegetable intake). Urine specimens will be collected at baseline visit (visit 2) and the follow-up visit (visit 6). Specimens will be labeled with SOTRUE ID labels and retrieved by Quest Diagnostics within 48hr via courier. Specimens will be stored at room temperature until retrieval.

If the participant discontinues study intervention early, we plan to encourage the participant to continue to come for all the follow-up visits.

7 <u>SAFETY ASSESSMENTS</u>

7.1 Specification of Safety Parameters

This is a dietary intervention study of typical foods consumed in participants' natural environment and thus poses minimal risk. Nevertheless, it is possible the participants could develop an adverse reaction to a new ingredient or have fluctuations in blood pressure based on sodium levels that might increase fall risk.

7.2 Methods and Timing of Assessing, Recording, and Analyzing Safety Parameters

Participants will be asked about any adverse food reactions and any fall history prior to feeding, 7-days into the study, and after the study is complete. We will also monitor blood pressure at day 10 of the study. We will use the following definitions of adverse events and serious adverse events.

7.3 Adverse Events and Serious Adverse Events

Adverse Event (AE): any untoward or unfavorable medical occurrence in a participant, temporally associated with the participants' involvement in research (i.e., after RV), whether or not considered related to participation in the research, including:

- New disease diagnosis or illness
- Worsening of pre-existing disease or illness
- New or worsening symptom or sign

Serious Adverse Event (SAE): an adverse event that meets 1 or more of these criteria:

- Results in death
- Requires or prolongs hospitalization
- Is life threatening
- Results in persistent or significant disability or incapacity
- Results in congenital abnormality or birth defect
- Is a condition which the investigator judges to represent a significant hazard

- Is a SOTRUE-specific serious adverse event:
  - A fall event
  - An allergic reaction
  - Extremely high or low blood pressure measurements or high pulse rate*
    - Systolic blood pressure >180 mm Hg
    - Systolic blood pressure < 90 mm Hg
    - Diastolic blood pressure >110 mm Hg
    - Diastolic blood pressure < 40 mm Hg
    - Pulse rate > 120 beats per minute

    *Low blood pressures may require discontinuation of the study intervention.

In the current project, no laboratory values are collected to assess safety.
During the screening, run-in, intervention day 10-13, and follow up visits participants will have their blood pressure checked. An algorithm will be followed by the research assistant for persons with abnormal blood pressures to refer them to appropriate healthcare, and to notify the study physician(s). At the baseline, telephone, and follow up visits information will be collected from participants about any adverse events, which will be reported to the study physician(s).

Solicited adverse events include uncomfortable fullness, hunger, bloating, constipation, diarrhea, excessive thirst, fatigue, headache, lightheadedness with standing, nausea, falls, falls with injury, allergic reactions (including food related), and any hospitalizations. Additionally, the participant may contact the study physician by phone in the event of a fall or serious adverse event (hospitalization).

All study visit data will be collected by the research assistant and then entered into a REDCap database. Data of clinical importance to eligibility or safety will be reviewed by a study physician. Study visits are scheduled frequently, and the duration of the intervention is 2 weeks, ensuring that any adverse event is reported and evaluated at intervals of about 1 week.

Random urine samples will be collected for sodium, potassium and creatinine at randomization and follow up visits to evaluate compliance with the study diet by the individual. No other laboratory testing will be done and no samples will be retained for future use. No safety lab tests will be done in this study.

Study staff are available on-site and via dedicated phone line for spontaneous reporting of adverse events by participants. In addition, we will monitor portion tolerance based on daily meal tracking forms.

7.3 Reporting Procedures

Pursuant to our ethical and regulatory responsibilities as well as to determine if further participation in the study is appropriate, we will provide participants contact information and request them to notify us of any new health issue or accident, particularly any falls. We will capture details of any adverse events occurring from the time of randomization to study completion or to document absence of adverse events during the study period.

If any AE or SAE is reported the research assistant will inform the study physician, who will determine if it is safe for the person to continue in the study.

Serious adverse events will be tracked until resolution or the end of the participant's involvement in the study. The principal investigator will report any SAE to the Hebrew SeniorLife IRB.

For extreme blood pressure measurements observed at any time during the study we will adhere to the following procedure:

High Values

- If SBP > 200:Notify SOTRUE Study Physician and advise participant to follow-up with primary care provider within 1 days (refer to emergency room if symptomatic)
- If SBP >180: Notify SOTRUE Study Physician and advise participant to follow-up with primary care provider within 3 days (same day if symptomatic)
- If SBP >160, but ≤180: Advise participant to follow up with primary care provider within 1 month
- If DBP > 120: Notify SOTRUE Study Physician and advise participant to follow-up with primary care provider within 1 days (refer to emergency room if symptomatic)
- If DBP > 110: Notify SOTRUE Study Physician and advise participant to follow-up with primary care provider within 3 days (same day if symptomatic)
- If DBP >100, but ≤110: Advise participant to follow up with primary care provider within 1 month
- If Pulse >120 bpm: Do not have participant participate in the TUG test and advise them to follow up with their primary care provider within 7 days

Low Values

- If SBP <90: Notify SOTRUE Study Physician and advise participant to follow-up with primary care provider within 7 days
- If DBP <40: Advise participant to follow up with primary care provider within 1 month

<u>Meal size concerns</u>
Participants with patterns of inadequate food quantity or conversely undereating due to the provision of too much food will be contacted for adjustments to their meal size. In particular, we will reach out to participants with known glucose impairments to ensure the appropriateness of their meal size with plans for adjustments based on feedback the preceding 3-4 days.

7.4 Safety Monitoring

This study maintains a committee of physicians that will meet as needed to discuss safety concerns. There is no formal DSMB.

7.5 Adverse Events and Serious Adverse Events

Adverse event (AE): any untoward or unfavorable medical occurrence in a participant, temporally associated with the participants' involvement in research (i.e., after RV), whether or not considered related to participation in the research, including:

- New disease diagnosis or illness
- Worsening of pre-existing disease or illness
- New or worsening symptom or sign

Serious adverse event (SAE): an adverse event that meets 1 or more of these criteria:

- Results in death
- Requires or prolongs hospitalization
- Is life threatening
- Results in persistent or significant disability or incapacity
- Results in congenital abnormality or birth defect
- Is a condition which the investigator judges to represent a significant hazard
- Is a SOTRUE-specific serious adverse event:
  - A fall event
  - An allergic reaction
  - Extremely high or low blood pressure measurements or high pulse rate*
    - Systolic blood pressure >180 mm Hg
    - Systolic blood pressure < 90 mm Hg
    - Diastolic blood pressure >110 mm Hg
    - Diastolic blood pressure < 40 mm Hg
    - Pulse rate > 120 beats per minute

      *Low blood pressures may require discontinuation of the study intervention.

In the current project, no laboratory values are collected to assess safety.

During the screening, run-in, intervention day 10-13, and follow up visits participants will have their blood pressure checked. An algorithm will be followed by the research assistant for persons with abnormal blood pressures to refer them to appropriate healthcare, and to notify the study physician(s). At the baseline, telephone, and follow up visits information will be collected from participants about any adverse events, which will be reported to the study physician(s).

Solicited adverse events include uncomfortable fullness, hunger, bloating, constipation, diarrhea, excessive thirst, fatigue, headache, lightheadedness with standing, nausea, falls, falls with injury, allergic reactions (including food related), and any hospitalizations. Additionally, the participant may contact the study physician by phone in the event of a fall or serious adverse event (hospitalization).

All study visit data will be collected by the research assistant and then entered into a REDCap database. Data of clinical importance to eligibility or safety will be reviewed by a study physician. Study visits are scheduled frequently, and the duration of the intervention is 2 weeks, ensuring that any adverse event is reported and evaluated at intervals of about 1 week.

Random urine samples will be collected for sodium and potassium at randomization and follow up visits to evaluate compliance with the study diet by the individual. No other laboratory testing will be done and no samples will be retained for future use. No safety lab tests will be done in this study.

7.6 Reporting Procedures

Pursuant to our ethical and regulatory responsibilities as well as to determine if further participation in the study is appropriate, we will provide participants contact information and request them to notify us of any new health issue or accident, particularly any falls. We will capture details of any adverse events occurring from the time of randomization to study completion or to document absence of adverse events during the study period.

If any AE or SAE is reported the research assistant will inform the study physician, who will determine if it is safe for the person to continue in the study.

Serious adverse events will be tracked until resolution or the end of the participant's involvement in the study. The principal investigator will report any SAE to the Hebrew SeniorLife IRB.

8 INTERVENTION DISCONTINUATION

Participation in this study is voluntary. If a participant discontinues the intervention, the participation in the follow-up of outcomes will be encouraged. If the participant is unable to come to any visits due to a conflict in their schedule, we will accommodate and try to complete their visit earlier or later to maximize the participation.

9 STATISTICAL CONSIDERATIONS

9.1 General Design Issues

This is an individual level, randomized, parallel-arm trial testing the primary hypothesis that a low sodium meal plan will reduced seated systolic blood pressure after about 14-days of feeding, compared to a typical sodium meal plan. For additional details please refer to our statistical analysis plan.

9.2 Sample Size and Randomization

To detect the difference in seated SBP observed in the DASH-Sodium trial[5] at 2-weeks (-9.53 mm Hg, standard deviation of 10.46, a type 1 error of 0.05 with 80% power), we would need 20 people in both arms (total 40). We will recruit 45 participants to account for a 10% attrition rate. Effect and variance data from the other endpoints will be used for sample size calculations in subsequent proposals.

9.2.1 Treatment Assignment Procedures

Participants will be randomized using a computer-generated randomization scheme with varying sized blocks to 1 of 2 sequences in a 1:1 ratio such that approximately half of participants will undergo the low sodium diet intervention, while the other half will undergo the usual sodium meal

plan intervention. Sex will be used as a stratification variable to ensure that sex is evenly distributed between interventions.

9.3 Interim analyses and Stopping Rules

Not applicable: Given the short duration of the study, no interim analyses have been planned.

9.4 Outcomes

9.4.1 Primary outcome

Seated systolic blood pressure measured during the follow-up visit.

9.4.2 Secondary outcomes

a. Seated diastolic blood pressure

b. Standing blood pressure

c. Orthostatic hypotension

d. Timed Up and Go Test

e. Urinary sodium, potassium, and creatinine excretion

f. Orthostatic symptoms

9.5 Data Analyses

Primary analysis

We will first confirm that the residuals of seated blood pressure are normally distributed (if not then it will be log-transformed). We will compare net effect of reduced sodium versus usual diet on 2-week seated blood pressure. This comparison will be performed by fitting a linear regression with adjustment for baseline seated blood pressure. We will perform an intent-to-treat analysis.

Our hypothesis is that 2-weeks of low sodium dietary intervention will lower seated blood pressure compared to 2-weeks of usual sodium diet. The null hypothesis is that there will be no difference in seated blood pressure between the two intervention arms. The comparison between the two treatment groups will be performed by comparing the regression coefficient for treatment with zero (two-sided Wald test at 0.05 significance level).

Secondary Pre-specified Subpopulations Analysis

We will repeat the primary analysis to compare subpopulations based on the following criteria:

1. Baseline antihypertensive use (yes vs no)
2. Baseline diabetes (yes vs no)
3. Sex (men or women)

We will fit general linear models including a subpopulation of interest as a main effect term and an interaction with treatment term. The interaction terms will be used to assess for effect modification by baseline characteristic. Note these analyses are contingent on having sufficient number of participants with and without baseline antihypertensive use. Other stratified or subpopulations analyses based on baseline characteristics may be performed for hypothesis generation beyond the main study.

Sensitivity analyses

1. Models from the analyses specified above will be fit with and without adjustment for baseline seated blood pressure.
2. We will perform three separate on-treatment analysis: (i) participants who ate 25% or less of non-study foods during the low sodium intervention, (ii) participants whose estimated calorie intake was met or exceeded by the food provided through the low sodium intervention, and (iii) persons who did not change antihypertensive medications during the post-randomization study period

Analyses of other outcomes

- **Compliance:** Compliance will be quantified by (i) counts and proportion of participants who ate 25% or less of non-study foods during the low sodium intervention, (ii) counts and proportion of participants whose estimated calorie intake was met or exceeded by the food provided through the low sodium intervention. The data will be reported by treatment group and overall.
- **Secondary Endpoints:** We will examine the effect of the low sodium diet on the following additional outcomes, testing the null hypothesis that there is no difference in the following outcomes between the low sodium diet and usual sodium diet intervention. Continuous outcomes will be evaluated using linear regression to compare change between the 2 interventions arms with and without adjustment for baseline. We will examine the distribution of all variables. We will use linear regression for normally distributed outcomes and logistic regression for binary outcomes. Continuous variables with non-normal residuals will be log-transformed with effects reported as a % change.

    - Seated diastolic blood pressure
        - Continuous variable, normal distribution
    - Standing blood pressure
        - Continuous variable, normal distribution
    - Orthostatic hypotension
        - Binary variable, binomial distribution
    - Timed Up and Go Test
        - Continuous variable, normal distribution
    - Urinary sodium, potassium, and creatinine excretion
        - These may require log-transformation; anticipate continuous variables, normal distribution
    - Orthostatic symptoms
        - Binary variables (severe vs not), binomial distribution

Handling of missing data: We will employ a number of recommended strategies to prevent missing data:

- A simplified data collection schedule that minimizes participant burden;
- Intention-to-treat analysis that includes following participants according to the data collection schedule regardless of compliance with the study intervention;
- Frequent engagement with the participants through visit reminder calls or notes;
- A 24-hour phone number that participants can contact for questions and support;
- Contiguous windows of time during which specific follow-up visits are allowed;
- Monetary incentives to encourage enrollment and continued participation;
- Rigorous training of clinic staff emphasizing the importance of
  - Positive and warm interpersonal relationships between the participants and study staff
  - Study commitment during the consent process to ensure that potential participants understand the importance of completing the study
  - Addressing participant concerns to minimize dissatisfaction
  - Collecting data even if a participant discontinues the study treatment
  - Reasons for any drop-outs will be documented.

We will perform the following sensitivity analyses using established methods for addressing missingness in clinical trials: multiple imputations, best and worst-case scenarios, and use of the drop out event as a study end-point.[9] We will compare the results from these approaches with primary analysis result's to assess the primary result's robustness to the effects of missing data. Further, we will compare the baseline characteristics of complete cases and participants with missing measures between the two assignments.

Safety outcomes

We will compare self-reported symptoms that are thought to potentially arise from the diets. The symptoms will be summarized by the counts and proportions of participants in each group and overall. The treatment groups will be compared with respect to safety outcomes by using a chi-square test (Fisher exact test when there are cell counts less than 5).

10 DATA COLLECTION AND QUALITY ASSURANCE

10.1 Data Collection Forms

Information will be collected for each participant by the research assistant, Ms. Abby Foley or the postdoctoral fellow, Dr. Courtney Millar, who will be blinded to the treatment groups. Each participant will be assigned a study ID and the records identifying participant name will be kept confidential in a locked study cabinet at the study clinic at Jack Satter House and, to the extent permitted by the applicable laws and/or regulations, will not be made publicly available. Urine samples collected will not identify the participant and will include only participant ID. Identifiers will be removed before entering the data electronically in a password protected REDCap database. The results of the study will only be published or presented as group data. No individual participants will be identified. Forms to collect data will be identified with a unique study number and kept locked in the study office. Once the data is entered into the electronic database, all source documents will be stored in a locked cabinet in PI's office at Marcus

Institute, HSL for a period of 7 years. After that the identifying information will be shredded.

Subject records may be reviewed in order to meet federal or state regulations. Reviewers may include the HSL Institutional Review Board, or others in order to meet regulatory requirements.

10.2 Data Management

Dr. Sahni will be responsible for the overall operation of the study, including IRB approvals, preparing budgets, monitoring expenses, data analysis and preparation of summary of findings. Dr. Juraschek will be responsible for training of study staff and reviewing data for any inconsistencies, reviewing eligibility, adverse events, and laboratory values and assisting Dr. Sahni with data interpretation and manuscript writing. Dr. Sahni and Juraschek will oversee the research assistant, Ms. Abby Foley, the postdoctoral fellow, Dr. Courtney Millar as well clinical dietitian, Ms. McNally.

Data will be managed with REDCap on a secure server. Embedded quality control procedures will be used to ensure data integrity. Ms. Foley, Ms McNally, or the postdoctoral fellow, Dr. Courtney Millar will collect all data, enter into REDCap database, work with Drs. Sahni and Juraschek to complete quality control processes. Data will be stored on a secure server behind HSL's firewall. Dr. Millar, Ms. Foley and Ms. McNally will fill complete the follow-up visit 1 and the completed forms will be kept in a locked cabinet at Jack Satter House by Ms. Foley until they are ready to be entered into REDCap. Once the data is entered electronically, all source documents will be kept in a locked cabinet in Dr. Sahni's office at the Marcus Institute. Safeguards to be used include keeping the source documents locked to protect participant privacy. We will also have a quality control process in place once the data is entered in REDCap. Dr. Juraschek will review the laboratory values of urine test and will also review the sources data for any inconsistencies. Data will be explored for potential outliers before the analysis.

10.3 Quality Assurance

10.3.1 Training

Describe types and mechanisms of training of staff for the study. Ms Foley and Dr. Sahni both participated in training at HSL in the TUG test procedure by a content expert. Subsequently both attended two extended practice sessions where each of them tried out each aspect of the procedure at the study site location.

Ms. Foley and Dr. Millar have completed an online training in administration of the MoCA standardized test.

Dr. Juraschek and Linda Godfrey-Bailey, an experienced clinical nurse specialist and research program manager, led Ms. Foley and Drs. Millar and Sahni through the process of obtaining height, weight, seated and orthostatic blood pressure measurements, with two practice sessions. Ms. Godfrey-Bailey also trained Dr. Millar and Ms. Foley in infection control practices including cleaning frequently used equipment, and collection and processing of urine samples. Ms. Foley also practiced use of all data collection forms under Drs. Sahni and Juraschek’s guidance.

10.3.2 Quality Control Committee

For this pilot study there is no quality control committee. Responsibility for oversight of quality control rests with the PI or designated co-investigator.

10.3.3 Protocol Deviations

In the event of a protocol deviation, Ms. Foley or Dr. Millar will report to Dr. Sahni with details of the event. The deviation report will be filed with the HSL IRB. In the event of possible injury to a participant from a study deviation the study physician will review the file and may follow up directly with the participant, and their primary care or other clinician to ensure referral for any care needed.

10.3.4 Monitoring

At each visit Ms. Foley or Dr. Millar will complete a summary checklist of all items to be done during the visit. Once consent has been obtained, Ms. Foley or Dr. Millar will add a check of consent at the start of each visit, prior to any data collection. Ms. Foley or Dr. Millar will review participant's data with Dr. Juraschek or Dr. Beach weekly to determine eligibility and once enrolled, participant safety.

No site monitoring will be done during the pilot.

11 PARTICIPANT RIGHTS AND CONFIDENTIALITY

11.1 Institutional Review Board (IRB) Review

This protocol and the informed consent document (Appendix 1) and any subsequent modifications will be reviewed and approved by the Hebrew SeniorLife IRB committee responsible for oversight of the study.

11.2 Informed Consent Forms

A signed consent form will be obtained from each participant. The consent form is only available in English at this time. The majority of residents at Jack Satter House are English speaking. At the request of a participant the research assistant will read the consent to them (for persons with visual difficulty or lower literacy). The consent form will describe the purpose of the study, the procedures to be followed, and the risks and benefits of participation. A copy will be offered to each participant and this fact will be documented in the participant's record.

11.3 Participant Confidentiality

All personal information obtained in the study will be kept confidential, and this information will only be available to the research staff. The records identifying the name of participants will be kept confidential in a locked study cabinet at the study clinic at Jack Satter House and, to the extent permitted by the applicable laws and/or regulations, will not be made publicly available.

To protect confidentiality, forms used to collect data will be identified with a unique study number and kept locked in the study office before entering the data electronically in a secure REDCap

database stored on the Hebrew SeniorLife servers. The results of the study will only be published or presented as group data. No individual participants will be identified.

Identifiable private information collected from participants during this study may be used for future research studies or shared with other researchers for future research. The identifiable private information may be used for future research of blood pressure management and falls. If the research investigator distributes information to other researchers or institutions, participant's identity will be concealed with a research code without identifiers so that they cannot be identified. No additional consent will be requested for the future use of participant's information. No biological specimens will be stored as part of this study.

Records may be reviewed in order to meet federal or state regulations. Reviewers may include representatives from the Study Sponsor and the HSL Institutional Review Board, or others in order to meet regulatory requirements. Protected health information may be reviewed by the clinical laboratory performing urine testing for this study. Information will not be released without written permission of the participant, except as necessary for monitoring by IRB, the FDA, the NIA, and the OHRP.

11.4 Study Discontinuation

The study may be discontinued at any time by the IRB, the Sponsor, the NIA, the OHRP, the FDA, or other government agencies as part of their duties to ensure that research participants are protected.

12 ETHICAL CONSIDERATIONS

This research project will follow the seven guiding ethical principles published by the NIH:

- Social and clinical value
- Scientific validity
- Fair subject selection
- Favorable risk-benefit ratio
- Independent review
- Informed consent
- Respect for potential and enrolled subjects

The intent is to answer the specific questions posed in this project using a scientifically valid method, with the collaboration of well-informed research participants who have been fairly selected. The risk versus benefit ratio in this project has been carefully reviewed by both internal and sponsor's independent review to maximize participant safety. The findings of this project will be disseminated via publication in scientific journals.

13 COMMITTEES

Oversight Committee: This committee will include the following personnel who will provide oversight for the study.

- **Shivani Sah**ni, PhD, Primary Investigator, Director, Nutrition Program and Associate Scientist, Hinda and Arthur Marcus Institute for Aging Research, Hebrew SeniorLife, Assistant Professor of Medicine, Beth Israel Deaconess Medical Center, Harvard Medical School
- **Stephen P. Juraschek**, PhD, MD, Co-Investigator, Assistant Professor of Medicine, Beth Israel Deaconess Medical Center, Harvard Medical School
- **Lewis Lips**itz, MD, Co-Investigator, Director, Hinda and Arthur Marcus Institute for Aging Research, Hebrew SeniorLife and Chief Academic Officer, Irving and Edyth S. Usen and Family Chair in Medical Research, Professor of Medicine, Harvard Medical School, Chief, Division of Gerontology, Beth Israel Deaconess Medical Center
- **Kenneth J. Mukamal, MD**, Co-Investigator, Internist, Department of Medicine, Beth Israel Deaconess Medical Center, Associate Professor of Medicine, Harvard Medical School, Visiting Scientist, Harvard TH Chan School of Public Health
- **Roger Davis, PhD**, Co-Investigator, Associate Professor of Medicine, Beth Israel Deaconess Medical Center

Medical Adjudication Committee: This committee will include the following physicians who will decide on the eligibility and safety issues related to the study.

- **Stephen P. Juraschek, PhD, MD,** Assistant Professor of Medicine, Beth Israel Deaconess Medical Center, Harvard Medical School
- **Jennifer L. Beach, MD**, Instructor in Medicine, Beth Israel Deaconess Medical Center, , Harvard Medical School

Dietetics and Culinary Nutrition Committee: This committee will include the following study members who will plan, prepare and dispense the dietary intervention at the Jack Satter House.

- **Shivani Sahni, PhD,** Primary Investigator, Director, Nutrition Program and Associate Scientist, Hinda and Arthur Marcus Institute for Aging Research, Hebrew SeniorLife, Assistant Professor of Medicine, Beth Israel Deaconess Medical Center, Harvard Medical School
- **Stephen P. Juraschek, PhD, MD,** Assistant Professor of Medicine, Beth Israel Deaconess Medical Center, Harvard Medical School
- **Misha Shtivelman,** Director of Culinary and Dietary Services at Hebrew SeniorLife and Jack Satter House
- **Alegria Cohen, MS, RD, LDN,** Dietitian at Hebrew SeniorLife
- **Robert Crevatis,** Head Chef, Jack Satter House

14 PUBLICATIONS AND RESEARCH FINDINGS

Publication of the results of this trial will be governed by the policies and procedures developed by the Steering Committee, and more generally by Harvard Medical School's Policy on Authorship. Any presentation, abstract, or manuscript will be made available for review by the sponsor and the NIA prior to submission.

15 REFERENCES

1. Losby JL, Patel D, Schuldt J, Hunt GS, Stracuzzi JC, Johnston Y. Sodium-Reduction Strategies for Meals Prepared for Older Adults. Journal of Public Health Management and Practice 2014;20:S23-S30.
2. Cogswell ME, Mugavero K, Bowman BA, Frieden TR. Dietary Sodium and Cardiovascular Disease Risk--Measurement Matters. N Engl J Med 2016;375(6):580-6.
3. Gangavati A, Hajjar I, Quach L, Jones RN, Kiely DK, Gagnon P, Lipsitz LA. Hypertension, orthostatic hypotension, and the risk of falls in a community-dwelling elderly population: the maintenance of balance, independent living, intellect, and zest in the elderly of Boston study. J Am Geriatr Soc 2011;59(3):383-9.
4. Pinto E. Blood pressure and ageing. Postgrad Med J 2007;83(976):109-14.
5. Weber MA, Neutel JM, Cheung DG. Hypertension in the aged: a pathophysiologic basis for treatment. Am J Cardiol 1989;63(16):25H-32H.
6. Juraschek SP, Daya N, Appel LJ, Miller ER, 3rd, Windham BG, Pompeii L, Griswold ME, Kucharska-Newton A, Selvin E. Orthostatic Hypotension in Middle-Age and Risk of Falls. Am J Hypertens 2017;30(2):188-95.
7. Juraschek SP, Daya N, Rawlings AM, Appel LJ, Miller ER, 3rd, Windham BG, Griswold ME, Heiss G, Selvin E. Association of History of Dizziness and Long-term Adverse Outcomes With Early vs Later Orthostatic Hypotension Assessment Times in Middle-aged Adults. JAMA Intern Med 2017;177(9):1316-23.
8. Juraschek SP, Miller ER, 3rd, Appel LJ. Orthostatic Hypotension and Symptoms in the AASK Trial. Am J Hypertens 2018;31(6):665-71.
9. Shen S, He T, Chu J, He J, Chen X. Uncontrolled hypertension and orthostatic hypotension in relation to standing balance in elderly hypertensive patients. Clin Interv Aging 2015;10:897-906.
10. de Bruïne ES, Reijnierse EM, Trappenburg MC, Pasma JH, de Vries OJ, Meskers CGM, Maier AB. Diminished Dynamic Physical Performance Is Associated With Orthostatic Hypotension in Geriatric Outpatients. J Geriatr Phys Ther 2018.
11. Kocyigit SE, Soysal P, Ates Bulut E, Isik AT. Malnutrition and Malnutrition Risk Can Be Associated with Systolic Orthostatic Hypotension in Older Adults. J Nutr Health Aging 2018;22(8):928-33.
12. El-Sayed H, Hainsworth R. Salt supplement increases plasma volume and orthostatic tolerance in patients with unexplained syncope. Heart 1996;75(2):134-40.
13. Mtinangi BL, Hainsworth R. Early effects of oral salt on plasma volume, orthostatic tolerance, and baroreceptor sensitivity in patients with syncope. Clin Auton Res 1998;8(4):231-5.
14. Cooper VL, Hainsworth R. Effects of dietary salt on orthostatic tolerance, blood pressure and baroreceptor sensitivity in patients with syncope. Clin Auton Res 2002;12(4):236-41.
15. Kranzler HR, Cardoni A. Sodium chloride treatment of antidepressant-induced orthostatic hypotension. J Clin Psychiatry 1988;49(9):366-8.
16. Gibbons CH, Schmidt P, Biaggioni I, Frazier-Mills C, Freeman R, Isaacson S, Karabin B, Kuritzky L, Lew M, Low P, et al. The recommendations of a consensus panel for the screening, diagnosis, and treatment of neurogenic orthostatic hypotension and associated supine hypertension. J Neurol 2017;264(8):1567-82.

17. Kerstens MN, Kobold ACM, Volmer M, Koerts J, Sluiter WJ, Dullaart RPF. Reference values for aldosterone-renin ratios in normotensive individuals and effect of changes in dietary sodium consumption. Clin Chem 2011;57(11):1607-11.
18. Fleg JL, Evans GW, Margolis KL, Barzilay J, Basile JN, Bigger JT, Cutler JA, Grimm R, Pedley C, Peterson K, et al. Orthostatic Hypotension in the ACCORD (Action to Control Cardiovascular Risk in Diabetes) Blood Pressure Trial: Prevalence, Incidence, and Prognostic Significance. Hypertension 2016;68(4):888-95.
19. Group SR, Wright JT, Jr., Williamson JD, Whelton PK, Snyder JK, Sink KM, Rocco MV, Reboussin DM, Rahman M, Oparil S, et al. A Randomized Trial of Intensive versus Standard Blood-Pressure Control. N Engl J Med 2015;373(22):2103-16.
20. Juraschek SP, Appel LJ, Miller ER, 3rd, Mukamal KJ, Lipsitz LA. Hypertension Treatment Effects on Orthostatic Hypotension and Its Relationship With Cardiovascular Disease. Hypertension 2018;72(4):986-93.
21. Whelton PK, Appel LJ, Espeland MA, Applegate WB, Ettinger WH, Kostis JB, Kumanyika S, Lacy CR, Johnson KC, Folmar S, et al. Sodium reduction and weight loss in the treatment of hypertension in older persons: a randomized controlled trial of nonpharmacologic interventions in the elderly (TONE). TONE Collaborative Research Group. JAMA 1998;279(11):839-46.
22. Wilke WL, Marley WS, Gotshall RW. Preconditioning with sodium deficits to improve orthostatic tolerance in rats. Aviat Space Environ Med 1995;66(8):757-62.
23. Davrath LR, Gotshall RW, Tucker A, Sadeh WZ, Luckasen GJ, Downes TR, Coonts CC. Moderate sodium restriction does not alter lower body negative pressure tolerance. Aviat Space Environ Med 1999;70(6):577-82.
24. Sacks FM, Svetkey LP, Vollmer WM, Appel LJ, Bray GA, Harsha D, Obarzanek E, Conlin PR, Miller ER, 3rd, Simons-Morton DG, et al. Effects on blood pressure of reduced dietary sodium and the Dietary Approaches to Stop Hypertension (DASH) diet. DASH-Sodium Collaborative Research Group. N Engl J Med 2001;344(1):3-10.
25. Peng AW, Appel LJ, Mueller NT, Tang O, Miller ER, 3rd, Juraschek SP. Effects of sodium intake on postural lightheadedness: Results from the DASH-sodium trial. J Clin Hypertens (Greenwich) 2019;21(3):355-62.
26. Jones DW, Luft FC, Whelton PK, Alderman MH, Hall JE, Peterson ED, Califf RM, McCarron DA. Can We End the Salt Wars With a Randomized Clinical Trial in a Controlled Environment? Hypertension 2018;72(1):10-1.
27. Effects of weight loss and sodium reduction intervention on blood pressure and hypertension incidence in overweight people with high-normal blood pressure. The Trials of Hypertension Prevention, phase II. The Trials of Hypertension Prevention Collaborative Research Group. Arch Intern Med 1997;157(6):657-67.
28. Whelton PK, Kumanyika SK, Cook NR, Cutler JA, Borhani NO, Hennekens CH, Kuller LH, Langford H, Jones DW, Satterfield S, et al. Efficacy of nonpharmacologic interventions in adults with high-normal blood pressure: results from phase 1 of the Trials of Hypertension Prevention. Trials of Hypertension Prevention Collaborative Research Group. Am J Clin Nutr 1997;65(2 Suppl):652S-60S.
