# Supplementary material for: The Effects of a Low Sodium Meal Plan on Blood Pressure in Older Adults: The SOTRUE Randomized Feasibility Trial
Source: Nutrients. 2021 Mar 16;13(3):964. doi: 10.3390/nu13030964 (PMC8002543; doi:10.3390/nu13030964)
Supplement: Supplementary file 1 [file nutrients-13-00964-s001.zip › Supplemental Material/Final SAP.pdf]

SOTRUE

STATISTICAL ANALYSIS PLAN

VERSION 1.0

October 29, 2019

Table of Contents

1 Introduction 3
Primary aim 3
Other aims 3
2 Data Source 4
3 Primary Outcome: Serum Uric Acid 5
Randomization and primary analysis 5
Power and sample size 5
Pre-specified Stratified Analysis 5
Sensitivity analyses 6
4 Other outcomes 6
Analysis 6
5 Missing data 8
6 Safety outcomes 9
7 References 10

1 Introduction

SOTRUE is an individual level, randomized feeding trial to determine the effect of 2 weeks of low sodium meal plan on seated blood pressure in 45 adults over the age of 60 years. Participants are randomly assigned to one of 2 meal plans: (1) Low sodium meal plan or (2) usual meal plan.

The **primary outcome** is the net effect of reduced sodium versus usual diet on 2-week seated blood pressure.

1.1 Primary aim

To determine the effects of a low sodium (<0.9 mg per kcal of energy intake**), 2-week meal plan compared with a usual meal plan (average sodium** ~2 mg per kcal of energy intake**) on seated BP among independently living older adults.** In this randomized controlled trial (RCT) of 45 patients, we will compare a low sodium diet intervention vs. usual sodium diet over 2 weeks to determine the effect of the sodium reduction on seated blood pressure.

1.2 Other aims

To estimate the effects of the low sodium diet on the following outcomes as well the variances of these outcomes:

A. Seated diastolic blood pressure
B. Standing blood pressure
C. Orthostatic hypotension (OH)
D. Timed Up and Go
E. Urinary sodium, potassium, and creatinine excretion
F. Orthostatic symptoms

2 Data Source

Data will be entered by clinic staff into a REDCap dataset. Meal nutrient information will be derived from the Computrition platform. Backup files of the database will be generated and stored at regular intervals in a secure, off-site location, to permit regeneration of the database in the event that it is destroyed.

3 Primary Outcome: Seated Blood Pressure

3.1 Randomization and primary analysis

Participants will be randomized using a computer-generated randomization scheme with varying sized blocks to 1 of 2 meal plans in a 1:1 ratio such that approximately half of participants will undergo the low sodium diet intervention, while the other half will undergo the usual sodium meal plan intervention. Sex will be used as a stratification variable to ensure that sex is evenly distributed between interventions.

We will first confirm that the residuals of seated blood pressure are normally distributed (if not then it will be log-transformed). We will compare net effect of reduced sodium versus usual diet on 2-week seated blood pressure. This comparison will be performed by fitting a linear regression model with adjustment for baseline seated blood pressure. We will perform an intent-to-treat analysis.

Our hypothesis is that 2-weeks of a low sodium meal plan intervention will lower seated blood pressure compared to 2-weeks of a usual sodium diet. The null hypothesis is that there will be no difference in seated blood pressure between the two intervention arms. The comparison between the two treatment groups will be performed by comparing the regression coefficient for treatment with zero (two-sided Wald test at 0.25 significance level to avoid type II error)[1] using linear regression with adjustment for baseline blood pressure.

Our primary outcome will be the average of two systolic blood pressures measured on days 12-15 of the study period. A sensitivity analysis will be performed examining the last systolic blood pressure measurement.

3.2 Power and sample size

To detect the 2-week difference in seated SBP (primary outcome) observed in the DASH-Sodium trial[2] (-9.53 mm Hg, standard deviation of 10.46, a type 1 error of 0.05 with 80% power), we need 20 people in each arm (total 40). We will recruit 45 participants to account for a 10% attrition rate. Effect size and variance for the other endpoints (especially OH) will be used to inform power calculations for subsequent proposals.

3.3 Secondary Pre-specified Subpopulations Analysis

We will repeat the primary analysis to compare subpopulations based on the following criteria:

1. Baseline antihypertensive use (yes vs no)
2. Baseline diabetes (yes vs no)
3. Sex (men or women)

We will fit general linear models including a subpopulation of interest as a main effect term and an interaction with treatment term. The interaction terms will be used to assess for effect modification by baseline characteristic. Note these analyses are contingent on having sufficient number of participants with and without baseline antihypertensive use.

Other stratified or subpopulations analyses based on baseline characteristics may be performed for hypothesis generation beyond the main study.

3.4 Sensitivity analyses

1. Models from the analyses specified above will be fit with and without adjustment for baseline seated blood pressure.
2. We will perform three separate on-treatment analyses: (i) participants who ate 25% or less of non-study foods during the low sodium intervention, (ii) participants whose estimated calorie intake was met or exceeded by the food provided through the low sodium intervention, and (iii) persons who did not change antihypertensive medications during the post-randomization study period
3. We will examine blood pressure (systolic and diastolic) using the last measurement alone.

4 Other outcomes

4.1 Compliance

Compliance will be quantified by (i) counts and proportion of participants who ate 75% or more of study foods. The data will be reported by treatment group and overall.

4.2 Secondary Endpoints

We will examine the effect of the low sodium diet on the following additional outcomes, testing the null hypothesis that there is no difference in the following outcomes between the low sodium diet and usual sodium diet intervention. We will examine the distribution of all variables. We will use linear regression for normally distributed outcomes and logistic regression for binary outcomes with and without adjustment for baseline. Continuous variables with non-normal residuals will be log-transformed with effects reported as a % change. If residuals are still not normally distributed even after log transformation we will apply non-parametric approaches.

A. Seated diastolic blood pressure (average of two measurements days 12 and 14)
   - Continuous variable, normal distribution; as a sensitivity we will also examine last diastolic blood pressure measurement alone

B. Standing blood pressure
   - Continuous variable, normal distribution

C. Orthostatic hypotension
    - Binary variable, binomial distribution

D. Timed Up and Go Test
    - Continuous variable, normal distribution

E. Urinary sodium concentration, potassium concentration, sodium/creatinine ratio, potassium/creatinine ratio, and sodium/potassium ratio
    - These may require log-transformation; anticipate continuous variables, normal distribution

F. Orthostatic symptoms
    - Binary variables (severe vs not), binomial distribution

G. Side effects (5-point Likert scale)
    - Depending on the distribution of responses, side effect data will be analyzed as a continuous variable or dichotomized (none or any symptoms)

5 Missing data

We will employ a number of recommended strategies to prevent missing data:

- A simplified data collection schedule that minimizes participant burden;
- Intention-to-treat analysis that includes following participants according to the data collection schedule regardless of compliance with the study intervention;
- Frequent engagement with the participants through visit reminder calls or notes;
- A 24-hour phone number that participants can contact for questions and support;
- Contiguous windows of time during which specific follow-up visits are allowed;
- Monetary incentives to encourage enrollment and continued participation;
- Rigorous training of clinic staff emphasizing the importance of
    - Positive and warm interpersonal relationships between the participants and study staff
    - Study commitment during the consent process to ensure that potential participants understand the importance of completing the study
    - Addressing participant concerns to minimize dissatisfaction
    - Collecting data even if a participant discontinues the study treatment
    - Reasons for any drop-outs will be documented.

We will perform the following sensitivity analyses using established methods for addressing missingness in clinical trials: multiple imputations, best and worst-case scenarios, and use of the drop out event as a study end-point.[3] We will compare the results from these approaches with primary analysis result's to assess the primary result’s robustness to the effects of missing data. Further, we will compare the baseline characteristics of complete cases and participants with missing measures between the two assignments.

6 Safety outcomes

We will compare self-reported symptoms that are thought to potentially arise from the diets. The symptoms will be summarized by the counts and proportions of participants in each group and overall. The treatment groups will be compared with respect to safety outcomes by using a chi-square test (Fisher exact test when there are cell counts less than 5).

7 References

1. Moore CG, Carter RE, Nietert PJ, Stewart PW. Recommendations for Planning Pilot Studies in Clinical and Translational Research. *Clin Transl Sci*. 2011;4(5):332-337. doi:10.1111/j.1752-8062.2011.00347.x

2. Sacks FM, Svetkey LP, Vollmer WM, et al. Effects on blood pressure of reduced dietary sodium and the Dietary Approaches to Stop Hypertension (DASH) diet. DASH-Sodium Collaborative Research Group. *N Engl J Med*. 2001;344(1):3-10. doi:10.1056/NEJM200101043440101

3. Shih W. Problems in dealing with missing data and informative censoring in clinical trials. *Curr Control Trials Cardiovasc Med*. 2002;3(1):4.
