# Supplementary material for: The Effects of a Low Sodium Meal Plan on Blood Pressure in Older Adults: The SOTRUE Randomized Feasibility Trial
Source: Nutrients. 2021 Mar 16;13(3):964. doi: 10.3390/nu13030964 (PMC8002543; doi:10.3390/nu13030964)
Supplement: Supplementary file 1 [file nutrients-13-00964-s001.zip › Supplemental Material/REV. Supplement Materials - Methods and Tables and Figures_MARKED.docx]

**Supplement Material**

**The effects of a low sodium meal plan on blood pressure in older adults: The SOTRUE randomized feasibility trial**

Stephen P Juraschek, MD, PhD,^1,2^ Courtney Millar, PhD,^2,3^ Abby Foley,^3^ Misha Shtivelman,^3,4^ Alegria Cohen MS RDN LDN,^3,4^ Virginia McNally MS RDN LDN,^3,4^ Robert Crevatis,^4^ Stephen M. Post MPA,^4^ Kenneth J Mukamal MD MPH,^1,2^ Lewis A. Lipsitz MD,^1,2,3^ Jennifer L Cluett MD,^1,2^ Roger B. Davis ScD,^1,2^ Shivani Sahni, PhD^2,3^

1. Beth Israel Deaconess Medical Center, Boston, Massachusetts
2. Harvard Medical School, Boston, Massachusetts
3. Hinda and Arthur Marcus Institute for Aging Research, Hebrew SeniorLife, Roslindale, Massachusetts
4. The Jack Satter House, Revere, Massachusetts

Author last names for PubMed indexing: Juraschek, Millar, Foley, Shtivelman, Cohen, McNally, Crevatis, Post, Mukamal, Lipsitz, Cluett, Davis, Sahni

Corresponding Author:

Stephen P Juraschek, MD PhD

Beth Israel Deaconess Medical Center

Division of General Medicine

Section for Research

330 Brookline Avenue, CO-1309, #216

Boston, MA 02215

Phone: 617-754-1416

Fax: 617-754-1440

E-Mail: sjurasch@bidmc.harvard.edu

**Supplement Material Contents**

**Supplement Methods SM1 & SM2, Figure SF1, & Tables ST1-ST5**

**Supplement Methods SM1.** Eligibility criteria.

**Supplement Methods SM2.** Detailed Methods for Non-primary Outcomes

**Supplement Figure SF1.** CONSORT diagram.

**Supplement Table ST1.** Table of assessments by study visit.

**Supplement Table ST2.** Effects of low versus typical sodium meals on participant-reported symptoms

**Supplement Table ST3.** Association of change in blood pressure from baseline with change in urine sodium or urine potassium

**Supplement Table ST4.** Effects of a low versus typical sodium meal plan on seated systolic blood pressure in pre-specified and exploratory subgroups

**Supplement Table ST5.** Sensitivity analyses of effects of a low versus typical sodium meal plan on seated systolic blood pressure

**Supplement Table ST6.** Self-reported dietary adherence and palatability according to dietary assignment.

**Supplement Methods SM1. Eligibility Criteria**

**Inclusion**

Eligible participants were residents of Jack Satter House, a congregate living facility operated by Hebrew SeniorLife and subsidized by HUD as part of its section 202 supportive housing for the elderly program. In addition, participants were required to be actively enrolled in Jack Satter House’s mandatory meal plan, age 60 years or older, have a resting SBP of 100-149 mmHg and DBP <100mmHg, and be on stable BP medications (no recent changes in the past 2 months or anticipated changes during the study period).

**Exclusion**

Exclusion criterion were cognitive impairment (defined by a Montreal Cognitive Assessment test <18), recent changes in BP medications in the past 2 months, terminal or mental illness, severe allergies to common foods, unwillingness to comply with either dietary assignment, physical inability to perform the TUG test, and history of active kidney dialysis or kidney transplant.

**Supplement Methods SM2. Detailed Methods for Non-primary Outcomes**

**Secondary Outcomes**

Pre-specified secondary outcomes were seated DBP, standing BP, orthostatic hypotension (OH), orthostatic symptoms, a TUG test, body mass index (BMI), urine sodium, urine potassium, urine creatinine, urine sodium-creatinine, and urine potassium-creatinine. DBP was measured concurrently with seated SBP detailed in the main text.

*Orthostatic Hypotension*

Standing BP was measured three times after 5 minutes of supine rest, beginning immediately after standing with each measurement separated by 30 seconds. Similar to seated assessments, participants were asked to empty their bladder and refrain from exercise, food, or caffeine for at least 30 minutes prior to measurement. OH was defined using the consensus definition based on the difference between standing and supine BP measurements as a drop in SBP of at least 20 mm Hg or a drop in DBP of at least 10 mm Hg.^1^ After standing, participants were also asked (yes or no) whether they felt dizzy, lightheaded, faint, or like they might black out in the process of standing or after standing up.

*Timed-Up-and-Go Test and Body Mass Index*

The average of two TUG tests was determined at baseline and after 2-weeks of feeding by recording the time it took each participant to stand up from a straight-backed chair, walk 3 meters in a straight line, and return to the seated position.^2^ BMI was derived from a calibrated scale and stadiometer at baseline and after 2 weeks of feeding.

*Urine measurements*

A urine specimen was collected at baseline and after 2 weeks of feeding. Specimens were stored in a sterile collection cup at room temperature and retrieved by Quest couriers within 24-hours of collection. Urine sodium (mmol/L), potassium (mmol/L), and creatinine (mg/dL) were determined via an ion selective electrode by Quest Diagnostics (Madison, New Jersey). Urine sodium-creatinine and potassium-creatinine ratios were derived by dividing either sodium or potassium concentration by creatinine concentration.

**Safety, Symptoms, Compliance, & Palatability**

At baseline, after 1 week of feeding, and after 2 weeks of feeding, participants were asked about falls or allergic symptoms in the preceding week. Participants were also asked to rate the frequency of the following symptoms using a Likert scale with 1 being never occurred and 5 being occurred nearly every day: uncomfortably full, hunger, bloating, constipation, diarrhea, excessive thirst, fatigue, headache, lightheadedness with standing, and nausea. Participants were also asked to quantify the average number of times they urinated while awake and the average number of times they would wake to urinate while sleeping at night.

Participants were asked about compliance and palatability after 1 week and 2 weeks of feeding with questions focused on: how likely they were to eat the diet long-term, how often they wasted or stored food because it was too much, or how often they supplemented foods with non-study foods because it was too little. For those who did not eat study foods, we asked them to describe the reason (disliked food, social reasons, spoiling, saving food for a later time, sharing with others, meal being lost/not delivered). Similarly, participants were asked to describe reasons for eating non-protocol foods (food preferences, social reasons, or wanting a break/more variety). All responses were provided on a 5-point Likert scale with 1 being none of the time/never and 5 being all of the time/always.

**Other covariates**

Other covariates of interest were self-reported: age (years), sex, race, hypertension status, hypertension medication use, history of OH, fall history (months since last fall), physical function (i.e., concerns about falling, bracing oneself, paused, or taking time during or after standing), presence of diabetes or related conditions (i.e., type 2 diabetes, gestational diabetes, prediabetes), cardiovascular disease, hyperlipidemia, gastrointestinal symptoms, cancer history, physical activity score (scale 0-42, calculated as [9*strenuous activity]+[5*moderate activity] adapted from Godin-Shephard Leisure Time Physical Activity Questionnaire),^3^ and alcohol use.

**References**

1. Kaufmann H. Consensus statement on the definition of orthostatic hypotension, pure autonomic failure and multiple system atrophy. *Clin Auton Res*. 1996;6(2):125-126.

2. Bennell K, Dobson F, Hinman R. Measures of physical performance assessments: Self-Paced Walk Test (SPWT), Stair Climb Test (SCT), Six-Minute Walk Test (6MWT), Chair Stand Test (CST), Timed Up & Go (TUG), Sock Test, Lift and Carry Test (LCT), and Car Task. *Arthritis Care Res (Hoboken)*. 2011;63 Suppl 11:S350-370. doi:10.1002/acr.20538

3. Amireault S, Godin G. The Godin-Shephard leisure-time physical activity questionnaire: validity evidence supporting its use for classifying healthy adults into active and insufficiently active categories. *Percept Mot Skills*. 2015;120(2):604-622. doi:10.2466/03.27.PMS.120v19x7

**Supplement Figure SF1. CONSORT Diagram**

**
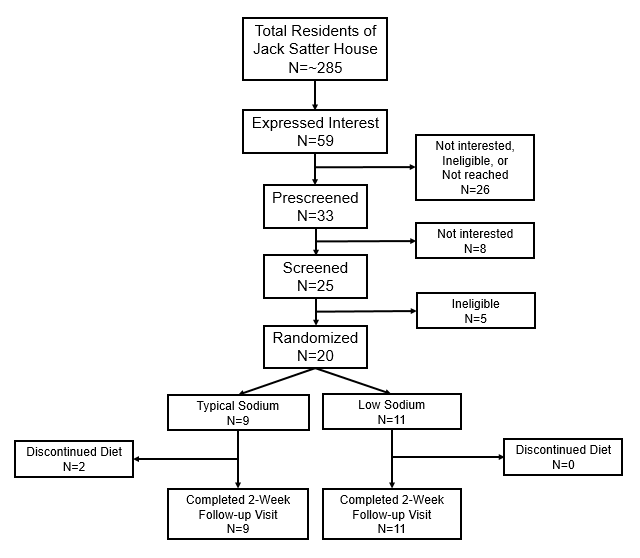
**

**Supplement Table ST1. Tables of assessments by visit**

| **Assessment** | **Prescreening (by phone)** | **Screening: Visit 1** | **Baseline, Enrollment, Randomization: Visit 2** | **Run In:**  **Visit 3** | **Treatment Visit 4 (Day 7)** | **Treatment Visit 5 (Day 10-13)** | **Follow-up: Final Visit 6**  **(Day 14)** |
| --- | --- | --- | --- | --- | --- | --- | --- |
| Medical History | **X** |  |  |  |  |  |  |
| MoCA |  | **X** |  |  |  |  |  |
| Seated blood pressure alone |  | **X** |  | **X** |  | **X** | **X** |
| Orthostatic hypotension |  | **X** |  |  |  |  | **X** |
| Informed Consent |  | **X** |  |  |  |  |  |
| Demographics |  | **X** |  |  |  |  |  |
| Physical Activity |  | **X** |  |  |  |  |  |
| Medications |  | **X** | **X** |  |  |  | **X** |
| Height |  | **X** |  |  |  |  |  |
| Weight |  | **X** | **X** |  |  |  | **X** |
| TUG |  |  | **X** |  |  |  | **X** |
| Urine collection |  |  | **X** |  |  |  | **X** |
| Compliance and Palatability |  |  |  |  | **X** |  | **X** |
| Symptoms/Adverse Events |  |  | **X** |  | **X** |  | **X** |
| Compliance Calendar |  |  |  |  | Each day of feeding | | |

| **Supplement Table ST2. Effects of low versus typical sodium meals on participant-reported symptoms, N=20 and N assessments=40** | | |
| --- | --- | --- |
|  | **Beta (95% CI)*** | ***P*** |
| Uncomfortably full | -0.37 (-1.25, 0.52) | 0.42 |
| Hunger | 0.22 (-0.94, 1.38) | 0.71 |
| Bloating | -0.12 (-0.75, 0.51) | 0.70 |
| Constipation | 0.03 (-0.61, 0.67) | 0.94 |
| Diarrhea | 0.32 (-0.02, 0.67) | 0.06** |
| Excessive thirst | -0.22 (-0.54, 0.10) | 0.18 |
| Fatigue | -0.10 (-0.81, 0.60) | 0.77 |
| Headache | -0.03 (-0.26, 0.20) | 0.80 |
| Lightheadedness with standing | -0.05 (-0.28, 0.17) | 0.64 |
| Nausea | 0.32 (-0.03, 0.67) | 0.07** |
| Number of times urinate while awake§ | 1.03 (-1.28, 3.34) | 0.38 |
| Number of times wake to urinate while sleeping at night§ | -0.05 (-0.52, 0.42) | 0.84 |
| Analyses performed using generalized estimating equations  *This represents the mean difference in Likert scale 1-5 from baseline for each symptom; 1 never occurred; 5 nearly every day | | |
| **While a *P*-value <0.05 was considered statistically significant, given the small sample of this pilot study, in our statistical analysis plan, a *P* ≤ 0.25 was considered noteworthy for discussion.  § N=19 and N of assessments=38 | | |

| **Supplement Table ST3. Association of change in blood pressure from baseline with change in urine sodium or urine potassium.** | | | | | | | |
| --- | --- | --- | --- | --- | --- | --- | --- |
|  | **Difference in SBP** | | |  | **Difference in DBP** | | |
| **Characteristic changed from baseline** | **β (95% CI)*** | ***P**** | ***r*** |  | **β (95% CI)*** | ***P**** | ***r*** |
| Log-transformed urine sodium | 10.93 (6.40,15.46) | <0.0001** | 0.34 |  | 5.94 (3.86, 8.01) | <0.0001** | 0.40 |
| Log-transformed urine sodium-creatinine ratio | 5.70 (2.67, 8.72) | 0.0003** | 0.30 |  | 2.91 (1.50, 4.32) | 0.0001** | 0.32 |
| Log-transformed urine potassium | -6.11 (-11.00,-1.23) | 0.015** | -0.29 |  | -0.97 (-3.29, 1.36) | 0.41 | -0.28 |
| Log-transformed urine potassium-creatinine ratio | -3.65 (-8.71, 1.40) | 0.16** | -0.16 |  | -0.01 (-2.39, 2.37) | 0.99 | -0.19 |
| *Adjusted for age, sex, and race. However, given that there was only 1 male and 1 black participant, this is better viewed as age-adjusted. | | | | | | | |
| ** While a *P*-value <0.05 was considered statistically significant, given the small sample of this pilot study, in our statistical analysis plan, a *P* ≤ 0.25 was considered noteworthy for discussion. | | | | | | | |

| **Supplement Table ST4. Pre-stated subgroup analyses** |  |  |  |
| --- | --- | --- | --- |
| **Systolic blood pressure, mmHg** | **Mean (95% CI)** | ***P*** | ***P*-interaction** |
| Entire sample (N=20) | -4.78 (-14.41, 4.85) | 0.31 | N/A |
| Women only (N=19) | -4.44 (-14.59, 5.71) | 0.37 | - |
| Baseline HTN medication use (N=12) | -12.92 (-25.64,-0.20) | 0.047** | 0.08** |
| No diabetic conditions (N=6) | -6.41 (-16.59, 3.78) | 0.19** | 0.86 |
| Calorie needs exceeded mean calories provided (N=16) | -7.78 (-18.77, 3.21) | 0.15** | 0.09** |
| Obesity (N=13)* | -8.78 (-22.09, 4.52) | 0.17** | 0.15** |
| Compliant group (N=8)* | -8.27 (-33.24,16.71) | 0.43 | 0.78 |
| *Note these were not pre-stated, but were performed for hypothesis generation. Compliance was defined as mean scores of 1 or 2, based on the responses to the following 2 Likert questions at 1 week and 2 weeks: “How often did you waste or store food because it was too much?” and “How often did you need to supplement foods with non-study foods because it was too little?” | | | |

** While a *P*-value <0.05 was considered statistically significant, given the small sample of this pilot study, in our statistical analysis plan, a *P* ≤ 0.25 was considered noteworthy for discussion.

| **Supplement Table ST5. Sensitivity analyses** |  |  |
| --- | --- | --- |
| **SBP Sensitivity analyses, N=20** | **Mean (95% CI)** | ***P*** |
| No baseline adjustment for SBP | -4.02 (-13.81, 5.77) | 0.40 |
| Restricted to the last 2 BP measurements | -5.52 (-15.37, 4.33) | 0.25* |
| Difference from baseline | -6.45 (-18.11, 5.20) | 0.26 |
| Repeat measures analysis with GEE | -4.81 (-13.12, 3.49) | 0.26 |
| Last visit only | -3.93 (-14.93, 7.07) | 0.46 |
|  |  |  |
| **Excluding the 2 subjects who discontinued meals, N=18** | **Mean (95% CI)** | ***P*** |
| Primary | -5.57 (-13.99, 2.85) | 0.18* |
| No baseline adjustment | -2.48 (-12.00, 7.04) | 0.59 |
| Restricted to the last 2 BP measurements | -5.68 (-14.70, 3.33) | 0.20* |
| Difference from baseline | -8.17 (-17.21, 0.87) | 0.07* |
| Repeat measures analysis with GEE | -5.50 (-12.75, 1.76) | 0.14* |
| Last visit only | -4.26 (-15.01, 6.49) | 0.41 |

* While a *P*-value <0.05 was considered statistically significant, given the small sample of this pilot study, in our statistical analysis plan, a *P* ≤ 0.25 was considered noteworthy for discussion.

| **Supplement Table ST6. Diet adherence and palatability** | |  |  |  |  |  |
| --- | --- | --- | --- | --- | --- | --- |
|  |  | **Typical Sodium** | |  | **Low Sodium** | |
|  |  | **N** | **Mean (SD)** |  | **N** | **Mean (SD)** |
| **I would eat this diet long-term** | | 9 | 1.9 (1.5) |  | 11 | 1.5 (0.9) |
| **How often did you waste or store food because it was too much** | | 7 | 1.6 (0.8) |  | 11 | 2.5 (1.0) |
| **How often did you need to supplement foods with non-study foods because it was too little** | | 7 | 2.0 (1.5) |  | 11 | 1.3 (0.6) |
| **Reasons for not eating food** | |  |  |  |  |  |
|  | Disliked food | 7 | 2.1 (1.1) |  | 11 | 1.7 (0.6) |
|  | Social reasons | 7 | 1.6 (1.5) |  | 11 | 1.4 (0.5) |
|  | It was spoiled or became spoiled | 7 | 1.3 (0.5) |  | 11 | 1.1 (0.3) |
|  | Saving food for a later time | 7 | 2.3 (1.1) |  | 11 | 1.5 (0.7) |
|  | Sharing with others | 7 | 1.0 (0.0) |  | 11 | 1.9 (1.4) |
|  | Meal was not delivered or was lost | 7 | 1.0 (0.0) |  | 11 | 1.3 (0.5) |
| **Reason for eating non-study foods** | |  |  |  |  |  |
|  | Preferred these foods to study meals | 7 | 1.3 (0.5) |  | 10 | 1.8 (1.2) |
|  | Social reasons | 7 | 1.7 (1.5) |  | 10 | 1.3 (0.5) |
|  | Something I liked was not included | 7 | 1.4 (0.8) |  | 10 | 1.8 (1.2) |
|  | Wanted a break or more variety | 7 | 1.0 (0.0) |  | 10 | 1.5 (1.1) |
| Likert scale ranging from 1 to 5 with 1 = none of the time/never; 5 = all of the time/always | |  |  |  |  |  |
